# Why are the youngest in class more often prescribed pharmacological treatment for ADHD?

**DOI:** 10.1007/s00148-026-01190-y

**Published:** 2026-07-23

**Authors:** Catia Nicodemo, Cheti Nicoletti, Joaquim Vidiella-Martin

**Affiliations:** 1 University of Oxford, Oxford, UK; 2 IZA, Bonn, Germany; 3 Brunel University of London, Uxbridge, UK; 4 University of York, York, UK; 5 ISER University of Essex, Colchester, UK; 6 Erasmus Centre for Health Economics Rotterdam (EsCHER), Erasmus University, Rotterdam, Netherlands

**Keywords:** Children, Mental health, School starting age, ADHD, England, I10, I20, J13

## Abstract

Children who start school at a younger age are more likely to receive pharmacological treatment for attention deficit hyperactivity disorder (ADHD). This pattern is widely attributed to peer-comparison biases, but the relative importance of different underlying mechanisms remains debated. We use administrative data from England to study how school starting age affects ADHD prescription rates from ages 5 to 15. Exploiting a sharp school-entry cutoff and comparing children born just before and after September 1, we estimate both age- and grade-specific effects of early school entry. We find that early starters are 40–50% more likely to be treated for ADHD by age 15. This long-term gap is entirely driven by first-time prescriptions among children aged 5 to 8. Using a novel decomposition strategy, we isolate the roles of relative age, absolute age, the length of school exposure, and age at school entry. We find that relative age is the primary long-term driver, aligning with previous evidence on peer-comparison bias, while absolute age and the length of school exposure have only short-lived effects. Our findings suggest that early starters are more likely to receive marginal diagnoses due to their relative immaturity, and we recommend refining diagnostic guidelines and classroom grouping practices to reduce diagnostic gaps.

**Supplementary Information:**

The online version contains supplementary material available at https://doi.org/10.1007/s00148-026-01190-y.

## Introduction

Attention deficit and hyperactivity disorder (ADHD) is a prevalent childhood disorder (Thomas et al. 2015), characterized by concentration problems, excessive activity, and impulsivity. It has been found to worsen educational attainment (Currie and Stabile 2006; Fletcher and Wolfe 2008; Ding et al. 2009; Currie et al. 2010) and income (Fletcher 2014) and increase crime (Fletcher and Wolfe 2009).

However, ADHD is difficult to diagnose, and there are increasing concerns about misdiagnoses. Children who start school at a relatively younger age than their school-grade peers are more likely to receive an ADHD diagnosis and medication than older peers. This difference has been attributed to their relative immaturity compared with older peers (Elder 2010). While both the oldest and the youngest children in a grade are likely to receive pharmacological treatment if they have extreme ADHD symptoms, only the youngest children are likely to be treated for milder symptoms (Persson et al. 2025). More marginal diagnoses for the relatively younger children imply an unfair and inefficient use of medical resources (Persson et al. 2025; Furzer et al. 2022).^1^ The treatment of marginally diagnosed patients may produce modest or even negative effects on their health and can have long-term economic costs.^2^

Quasi-experimental evidence has exploited school-entry age cutoffs to document the gap in ADHD diagnosis and pharmacological treatment rates for relatively younger children.^3^ For example, in England, the cutoff date is September 1, and there is a gap of almost 1 year in the age at which a child starts school if born just after September 1 (late school starters) rather than just before (early school starters). In this paper, we use rich administrative data from England to study what explains the gap in ADHD pharmacological treatment rates between early and late school starters.

In line with previous studies, we confirm a discontinuity in treatment rates at the school entry cutoff across all ages 5 to 15. However, when we look at the first-time prescription rate (i.e., the incidence of ADHD pharmacological treatments instead of their prevalence), we find a discontinuity at ages 5–8, but not after age 9. These findings suggest that the long-term gap in ADHD pharmacological treatment rates between early and late starters is caused by prescriptions initiated in the first years of primary school, highlighting the importance of accurate diagnosis in early primary school.

This gap is often attributed to teachers’ peer-comparison bias. Early starters are the youngest in their grade, so teachers may misinterpret their more immature behavior relative to older classmates (e.g., inattention or disruptiveness) as ADHD symptoms.^4^ Few studies have examined other mechanisms. Evans et al. (2010) provide suggestive evidence that the gap in ADHD diagnosis between early and late school starters is not explained by differences in stress and the length of school exposure by age. Schwandt and Wuppermann (2016) emphasize that the gaps in ADHD diagnosis and prescriptions between early and late school starters can partly be explained by differences in absolute age and the length of school exposure. We build on these previous papers and introduce a more comprehensive framework. This allows us to rule out some of these potential mechanisms and confirm that relative age is the main mechanism explaining the long-term effect.

Previous evidence has documented the effects of relative age and teacher comparison biases. Elder and Lubotsky (2009) show that the classmates’ average age at school entry increases the probability of a child being diagnosed with ADHD even after controlling for the child’s age at school entry, suggesting a potential relative age effect. Elder (2010) and Furzer et al. (2022) show that the symptoms of ADHD among children, as reported by teachers’ reports, plotted against the date of birth have a discontinuity at the cutoff date for school entry. In contrast, there is no discontinuity in parents’ reports.^5^

However, previous papers largely overlook other influences on teachers’ assessments beyond relative age. Teachers can have an amplified perception of ADHD symptoms in early starters, not only due to age but also because stress may temporarily worsen symptoms. Although school stress does not cause ADHD, which is primarily genetic (Riglin et al. 2016), it can temporarily worsen symptoms in class, prompting more teacher-reported symptoms and marginal diagnoses.^6^

Early starters may face more stress at school entry due to their lower age and maturity at that time—we refer to this as the *age at start of school* effect. They are also in a higher school grade than late starters at the same age, thus exposed to higher expectations and demands from teachers, which can increase their stress—*length of school exposure* effect. Young-for-grade children are more likely to experience an increase in stress caused by comparing themselves with classmates who tend to be older and more skilled (Kiessling and Norris 2023) and by an increased likelihood of being targeted for bullying because their behavior can stand out more (Sarzosa and Urzúa 2021)—*relative age* effect. Furthermore, the ADHD gap in pharmacological treatment between early and later starters may be underestimated if we do not account for the *absolute age* effect. Because rates of ADHD pharmacological treatment increase with age, a non-age-adjusted rate of ADHD for early starters who are observed at a younger age than late starters in the same grade could underestimate the actual rate.

Our conceptual framework considers four potential mechanisms behind the ADHD treatment gap between early and late starters: the age at the start of school, the length of school exposure, relative age, and absolute age. Unlike previous papers that focused solely on relative age, we adopt a model that includes these four mechanisms to explain the effect of an early school start on the rate of ADHD pharmacological treatment.^7^ Below, we explain how we address collinearity between absolute age, age at the start of school, length of school exposure, and relative age.

As in Black et al. (2011), we account for the effect of absolute age by observing both early and late starters at the same age, using administrative data on general practices to determine whether a child has been prescribed pharmacological treatment for ADHD at any point in their life. To disentangle the three remaining mechanisms, we follow Crawford et al. (2014) and compare the effect of starting school early for children observed at the same age with the corresponding effect for children in the same school grade. This comparison allows us to isolate the effects of absolute age and school exposure duration, revealing that both are short-lived and disappear by age 8. The only longer-term effects are thus the age at the start of school and the relative age. Starting school can cause more stress for early starters. However, we provide suggestive evidence that role of stress is, at most, minor in explaining the long-term effects by showing that starting school early only has small effects on anxiety diagnoses and drug prescriptions, which should be affected by manifestations of stress (Rockhill et al. 2010; Chaby et al. 2015) and is a common psychiatric comorbidity of ADHD (Toole and Frank 2024).^8^ Therefore, our results suggest that the long-term effect on ADHD pharmacological treatment rates is mainly (although not necessarily exclusively) driven by the relative age effect, as claimed in previous studies (see, e.g., Elder, 2010; Evans et al., 2010; Persson et al., 2025; Furzer et al., 2022; Bertoni et al., 2023).

We leverage novel administrative data from general practices and hospitals in England that provide information on children’s health conditions, prescriptions, and treatments from birth onward. Focusing on a cohort of children born between 2002 and 2010, we estimate the effect of starting school early on pharmacological treatment for ADHD at (i) each age between 5 and 15 and (ii) each grade from reception (kindergarten) to grade 10. Using the September 1 entry cutoff, we compare July–August to September–October births, controlling for observed child/family characteristics and medical practice fixed effects.^9^ The gap in ADHD pharmacological treatment rates between early and late starters does not decrease with age. By the end of compulsory schooling, early starters still have a prescription rate for ADHD 40-50% larger than late starters.^10^ This gap is mainly explained by differences in relative age and is driven by first-time prescriptions initiated in the first years of primary school.

We rule out the possibility that our estimates are biased by red-shirting practices or grade retention, as these practices are rare in England, unlike in the US (Crawford et al. 2013). Even in the presence of red-shirting, our estimate likely represents a lower bound on the true effect, since parents tend to delay entry for less mature children. We also rule out that our results are driven by the season of birth, as similar gaps have been documented across countries with varying cutoffs and in both hemispheres (Bedard and Dhuey 2006). Furthermore, our main findings are confirmed when considering the regression discontinuity approach and other sensitivity analyses.

Our first contribution to the literature is a broader understanding of the mechanisms, beyond relative age, underlying the gap in pharmacological treatment for ADHD between early and late starters. We analyze the effect of early start on ADHD pharmacological treatment rates across age and school grades and provide a conceptual framework to explain how comparing these rates helps identify the roles of each mechanism.

Our second contribution is to the emerging literature on marginal treatments (e.g., Einav et al., 2020; Persson et al., 2025; Currie and Zwiers, 2025; Bos et al., 2023). A gap in marginal treatments between early and late starters implies inefficient and unequal resource use. We use incidence and prevalence rates of ADHD pharmacological treatments across ages and grades to identify when marginal treatment gaps are most likely to emerge. This is important because ADHD pharmacological treatments, like other mental health treatments, tend to last for years, so initiating marginal treatments can have lasting effects on health and public spending.

Finally, we provide guidance on interventions to reduce the early-late starter treatment gap. We caution against indiscriminate red-shirting for all early school starters and suggest two alternatives to reduce marginal diagnoses among early starters: sorting children into classes based on age and improving diagnostic decision-making in early primary school.

The paper proceeds as follows: Section 2 outlines the conceptual framework. Section 3 reviews ADHD diagnosis, treatment, and the education system in England. Section 4 describes the data, and Section 5 details the identification strategy. Section 6 presents the main results on prevalence and incidence gaps, underlying mechanisms, initiation periods, and robustness checks. Section 7 discusses the interpretation of our results. Finally, Section 8 concludes with policy recommendations.

## Conceptual framework

Twin studies have found that ADHD is a highly heritable disorder (Jepsen and Michel 2006), but environmental factors can alter the severity of ADHD symptoms (Livingstone et al. 2016; Björkenstam et al. 2018; Hartman et al. 2019). School entry represents a transition into a more structured and evaluative environment, which may differentially affect children depending on developmental maturity. Evidence from the developmental psychology and education literatures suggests that transitions into structured group settings can be more demanding for younger or less mature children, suggesting that adjustment challenges or stress at entry could temporarily affect observed behavior (Rimm-Kaufman and Pianta 2000; Dettling et al. 1999; Ahnert et al. 2004; Gunnar and Quevedo 2007).^11^

Besides stress, another factor that may explain the gap in ADHD pharmacological treatment rates is a peer-comparison bias. As emphasized by Elder and Lubotsky (2009); McEwan and Shapiro (2008), and Elder (2010), early starters are younger and more immature than their classmates, so teachers may perceive ADHD symptoms in early starters as worse.

The temporary amplification and the magnified perception of ADHD symptoms caused by the heightened school stress and peer-comparison bias can lead to marginal diagnoses for early starters—i.e., to a higher probability of being diagnosed with ADHD even if the symptoms are moderate. As emphasized by Persson et al. (2025), these may result in prescriptions that increase healthcare costs without corresponding health improvements. Even if marginal treatments for early starters were beneficial, we would still face a misallocation of health resources, resulting in unfair undertreatment of late starters.

As explained in Section 1, there are four mechanisms through which being an early starter may affect ADHD diagnoses and prescriptions, which we describe in more detail in the following:

1. *Age at school entry*: Younger children have a lower level of emotional and cognitive maturity (low school readiness), which may be associated with greater short-run adjustment challenges when entering formal schooling, especially in the first school years (Rimm-Kaufman and Pianta 2000; Gunnar and Quevedo 2007). Stress, in this context, reflects differences in children’s capacity to cope with a given stimulus at a fixed point in time.
2. *Length of school exposure*: Early starters observed at the same age as late starters will have been exposed to more schooling and less preschool childcare. Teachers’ expectations and curriculum demands increase with school grades (length of school exposure), leading to an increase in school stress for early starters, which can amplify the severity of ADHD symptoms, especially in the school environment. Under this mechanism, stress does not arise simply because children are younger at a given point in time, but because prolonged exposure to the school environment increases the frequency and duration of situations in which their behavior is evaluated. In the early school years, early starters are placed in higher grades relative to their age and therefore face more demanding curricula and behavioral expectations, increasing the likelihood that ADHD-related behaviors are noticed and acted upon.^12^ Over time, however, continued exposure to schooling may also foster the development of socio-emotional skills and greater familiarity with classroom routines, which can attenuate these effects as children adapt (Cornelissen and Dustmann 2019). We therefore treat the net short-run effect of the length of school exposure as potentially positive, while allowing the data to determine whether this effect persists into later grades.
3. *Relative age*: Early starters are the youngest in their grade and can be perceived by teachers as having more severe symptoms due to peer-comparison bias. Furthermore, the stress caused by relative immaturity can worsen their mental health and temporarily aggravate their ADHD symptoms.^13^ Relative age is measured as the number of days between a child’s date of birth and the school-entry cutoff date. With a September 1 cutoff in England, children born on August 31 are the youngest in their cohort (relative age minus 364 days), while children born on September 1 are the oldest (relative age 0). Under this definition, more negative relative age values indicate being younger than classmates.
4. *Absolute age* (also known as age at test, i.e., age when ADHD is measured): Early starters have a lower absolute age than late starters in the same grade. The effect of absolute age is explained neither by stress nor by the peer comparison bias but by the fact that ADHD diagnoses and prescriptions increase steadily from age 5 to 15 (Scahill and Schwab-Stone 2000). If early and late starters are compared at the same time, late starters will be older and, hence, more likely to be diagnosed and treated.

If the main cause of the gap in ADHD pharmacological treatment rates between early and late starters was the length of school exposure and, therefore, the stress caused by higher schools’ and teachers’ expectations by grade, then a solution could be to adjust the curriculum to the needs of each child’s age within a grade. If the main driver of the gap was the age at school entry (i.e., the stress caused by lack of school readiness), then a solution could be to increase the school entry age. If, instead, the gap in ADHD pharmacological treatment rates were mainly explained by relative age, a better solution would be to group children within the same grade into more refined age-based classes or to improve diagnostic procedures to account for relative age. Finally, if the main mechanism behind the ADHD pharmacological treatment rates was the difference in absolute age between early and late starters, no intervention would be needed, since comparisons at the same age remove the gap. Below, we present a model that accounts for all four mechanisms.

We define a dummy variable $ADHD_{i,t}$ which takes value 1 if the child *i* received at least one prescription for ADHD in the 1-year period *t* and 0 otherwise, and we model the relationship between $ADHD_{i,t}$ and the four mechanisms discussed above adopting a linear probability model^14^ The assumption of a linear probability model will be relaxed in our sensitivity analysis.

1 $$\begin{aligned} \begin{aligned} ADHD_{i,t}&= \alpha _t + \gamma _{EXP,t} \, Exposure_{i,t} + \gamma _{AGEE,t} \,Age Entry_{i,t} \\&+ \gamma _{RELAGE,t} \, RelativeAge_{i,t} + \gamma _{ABSAGE,t} \,AbsAge_{i,t}+u_{i,t}, \end{aligned} \end{aligned}$$

where the subscript *t* denotes the specific 1-year interval in the life of child *i* (either age or school grade). The length of school exposure (*Exposure*), absolute age (*AbsAge*), age at school entry (*AgeEntry*), and relative age (*RelativeAge*) are measured at a daily level using exact dates. $u_{i,t}$ captures the effect of the covariates, which we will introduce in our empirical section, and a residual error term. Based on the mechanisms above, the probability of ADHD treatment should increase with the length of school exposure and absolute age, but decrease with age at entry and relative age. In other words, we expect $\gamma _{EXP,t}>0$, $\gamma _{ABSAGE,t}>0$, $\gamma _{RELAGE,t}<0$ and $\gamma _{AGEE,t}<0$.

Note that we allow the coefficients of the model to change across different periods in the child’s life, so our assumption of linearity in *Exposure*, *AbsAge*, *AgeEntry*, and *RelativeAge*, needs to hold only within each interval *t*. We also consider interactions between these mechanisms. For example, we allow the effects of relative age exposure and age at school entry to decrease as the child ages by allowing these effects to differ across *t* (age or school grade).

The effect of being an early school starter (born in July and August) with respect to late starters (born in September and October) is equivalent to the effect of increasing *Exposure* by approximately 1 year and decreasing *AgeEntry*, *RelativeAge*, and *AbsAge* by approximately 1 year, so the effect of being an early starter is given by the following:

2 $$\begin{aligned} \gamma _{t}= \gamma _{EXP,t} - \gamma _{AGEE,t} - \gamma _{RELAGE,t} - \gamma _{ABSAGE,t}. \end{aligned}$$

The slope coefficients in Eq. 1 cannot be separately identified because of multicollinearity. However, by observing both early and late starters in the same academic year, i.e., defining the time *t* as school grade *g*, we keep *Exposure* constant and identify the following:

3 $$\begin{aligned} \gamma _{g} = - \gamma _{AGEE,g} - \gamma _{RELAGE,g} - \gamma _{ABSAGE,g}, \end{aligned}$$

where $\gamma _{g}$ denotes the effect of being an early starter in academic grade *g*, which we call the same-grade effect. Similarly, if we observe children at the same age t=a, we ensure that *AbsAge* is the same for early and late starters, and we can identify the following effect of being an early starter at age *a*:

4 $$\begin{aligned} \gamma _a = \gamma _{EXP,a} - \gamma _{AGEE,a} - \gamma _{RELAGE,a}, \end{aligned}$$

which we call the same-age effect.

We compute $\gamma _a$ and $\gamma _g$ for age *a* going from 5 to 15 and for school grade *g* going from 0 (reception year/kindergarten)^15^ to 10, and we compare the same school grade effect $\gamma _g$ with the same age effect $\gamma _a$ computed at a=g+5, where 5 is the age that children turn during their first year in primary school in England, implying that $\gamma _{AGEE,a}=\gamma _{AGEE,g}$ and $\gamma _{RELAGE,a}=\gamma _{AGEE,g}$. Therefore, by taking the difference between $\gamma _a$ and $\gamma _g$, we isolate the combined effect of the length of school exposure and absolute age:

5 $$\begin{aligned} \gamma _a - \gamma _{g} = \gamma _{EXP,a} + \gamma _{ABSAGE,g}. \end{aligned}$$

The comparison between the same-age and same-grade effects isolates the combined contribution of the length of school exposure and absolute age, $\gamma _{EXP,a} + \gamma _{ABSAGE,g}$. For ease of reference, Appendix Table A.1 summarizes the mapping between the coefficients in the conceptual framework and the effects estimated in the empirical analysis. Under the plausible assumption that both the absolute-age component (reflecting increasing diagnosis with age) and the exposure component (reflecting higher institutional demands at higher grades) operate in the same direction, a near-zero difference would suggest that these components are small in magnitude relative to the remaining mechanisms: relative age and age at entry. We expect both effects to be positive: absolute age, because treatment increases with age, and the length of school exposure, because early starters face more pressure from advanced curricula at least in the early school grades.

## Background

### ADHD: diagnosis and treatment

ADHD is one of the most common neurodevelopmental disorders during childhood (Mannuzza and Klein 2000). It is characterized by inattentiveness, hyperactivity, and impulsiveness. Most children are diagnosed when they are between 6 and 12 years old, and ADHD often persists into adulthood.

In England, when parents or teachers notice ADHD symptoms in a child, they are advised to raise their concerns with the child’s school’s special educational needs coordinator or a general practitioner (GP). GPs cannot diagnose ADHD; they can discuss parental concerns and refer the child to a specialist evaluation if necessary. Several specialists can conduct a formal assessment, including a psychiatrist, pediatrician, and community pediatrician,^16^ learning disability specialist, social worker, or occupational therapist with experience in ADHD diagnosis.

ADHD diagnosis is not determined through a single test. The process typically includes physical examinations and interviews with the child and significant others (such as parents and teachers). Symptoms must be consistently present for at least 6 months for a diagnosis to be made. These symptoms should be present in at least two different settings (which, in practice, involves showing them both at home and at school) to ensure that they are not a reaction to a particular teacher or parent. Therefore, the views of parents and teachers are crucial in determining a diagnosis.

In most cases, ADHD is treated with a combination of behavioral therapy and medication. For children below the age of 5, behavioral therapy, particularly training for parents, is recommended as the first line of treatment before any medication is tried. There are five types of drugs licensed for the treatment of ADHD in England: methylphenidate, lisdexamfetamine, dexamfetamine, atomoxetine, and guanfacine. While these drugs may not provide a permanent cure for the disorder, they can provide relief from some of the associated symptoms. They have been shown to improve concentration, reduce impulsivity, induce a sense of calm, and facilitate the learning and application of new skills in individuals with this condition. This treatment has the potential to aid children with ADHD in focusing better in the classroom and minimizing risky behaviors outside of school. Aizer (2008); Dalsgaard et al. (2014b); Chorniy and Kitashima (2016) show that children diagnosed with ADHD in pharmacological treatment have fewer hospital contacts if treated and that treatment, to some extent, protects against their engagement in criminal behavior. However, psychoactive medications also alter brain function and could have short- and long-term negative effects on the formation of human capital (Gould et al. 2009; Cascade et al. 2010; Currie et al. 2014).

The National Institute for Health and Care Excellence in the UK (NICE) provides guidelines for diagnosing and treating ADHD. Since 2008, the importance of considering relative age biases is explicitly included in these guidelines: “ADHD should be considered in all age groups, with symptom criteria adjusted for age-appropriate changes in behaviour” (NICE 2018).

### Early schooling in England

In England, children are supposed to start their first primary school year (which is equivalent to the first grade in elementary school in the US) in September after they turn 5, with a large majority of children starting schooling 1 year earlier and attending reception year, which is equivalent to the US kindergarten year. As a result, most children start school full-time in September after their fourth birthday, creating an almost 1-year age difference between children born just before and after September 1. Red-shirting (i.e., delaying a child’s entry to school) is uncommon in England, and virtually all children attend reception classes. We cannot observe the actual age at school entry in our administrative data, but Crawford et al. (2013) show that over 99% of children were enrolled in the correct academic year for their age when considering the full population of children in state (public) schools in 2008 in England, while Cornelissen and Dustmann (2019) document that 96% of pupils attended a reception year when considering children born between 2000 and 2001 in England. Parents can delay the start of reception class from September to later in the school year, but not beyond September after the child’s 5th birthday. This means that children who are delayed will still start the first grade in September after their 5th birthday. Furthermore, children born between April 1 and August 31 can apply to skip reception class, but they still start their first grade in September after their 5th birthday. All these delays are not automatic and require the parents to submit a successful application to the competent admission authority.^17^

Although most primary schools in England have a single cutoff date for school entry (September 1), in the very early 2000s, a few schools allowed children to start at different times during the academic year (for more details, see Cornelissen and Dustmann (2019)). Our sample covers children who started school between 2007 and 2015 (born between 2002 and 2010). During this period, almost all minority schools switched to a single-entry cutoff date.^18^

Before starting reception class, children in England can be in formal childcare or be cared for at home. All 3- and 4-year-olds have the right to fully subsidized part-time preschool, which provides approximately 12.5 h of free childcare per week (Blanden et al. 2016).

Children are assessed at the start and end of the reception year with an on-entry assessment (the reception baseline assessment) and a progress assessment (summary assessment). After completing the reception class, children attend primary (elementary) school from ages 5 to 11 and secondary school from 11 to 16. Most schools in England are state schools that do not charge tuition fees, and fewer than 10% of children are enrolled in independent schools requiring tuition fees. The Standard Attainment Tests (SATs) are administered at ages 7, 11, and 14, and the General Certificate of Secondary Education (GCSE) is typically taken at ages 15–16. Compulsory schooling ends at age 16.^19^

## Data

### Data sources

We use data from QResearch, a large consolidated database derived from anonymized health records from general practices in England, matched with hospital administrative data, the Hospital Episode Statistics (HES). In our analysis, we use individual-level information on general practice diagnostics, drug prescriptions, and maternity records from HES, enabling us to link children to their mothers. QResearch has been used as a representative sample of the primary care population in well-published medical journals (Hippisley-Cox and Coupland 2010; Gao et al. 2021; Aveyard et al. 2021).^20^

For all children in the data, we consider their prescriptions for ADHD-related disorders^21^ and a vector of sociodemographic characteristics which includes the region of residence, ethnicity, general practice identifier, and socioeconomic status (SES). SES is measured at the postal code level using the Townsend deprivation index, reported in quintiles (Coupland et al. 2007). The Townsend deprivation index is an area-based measure of deprivation (Townsend et al. 1988) which is constructed from the following four census variables: households without a car, overcrowded households, households not owner-occupied, and unemployed individuals.^22^ Our data also include information on the health at birth of children, including maternal age at birth, birth weight, and date of birth. We observe the dates of entry and exit from maternity care and use the median between these two dates as a proxy for the date of birth. The average length of stay in maternity care is 2 days. In Section 6.4, we run several sensitivity analyses to show that this approximation of the date of birth does not bias our results.

In addition to information on ADHD diagnoses and prescriptions, QResearch and the linked Hospital Episode Statistics provide rich health information for both children and mothers that we use in internal validity and robustness checks. These data include hospital admissions for a range of pediatric and maternal conditions recorded in HES, as well as primary care records containing information on maternal health behaviors such as smoking status and body mass index (BMI). The timing and construction of these variables, and their use in balance tests, are described in Section 6.1 for maternal characteristics and in Section 6.4 for child placebo health outcomes.

### Main sample and variables

Our main sample comprises all singletons born between July 1 and October 30 in any of the years between 2002 and 2010, and traceable in the administrative data up to age 10 (N=97,117). As detailed in Section 3, almost all children in England start the school reception (kindergarten) year in September after their fourth birthday. Restricting the sample to children born between July and October ensures that children are comparable in most dimensions, except for their school-starting age. We exclude individuals with missing information on socioeconomic variables (419 observations, less than 0.5% of the initial sample). This results in a final sample of 96,698 children. Since the children in our sample were born between 2002 and 2010, and our data extends to the end of 2020, we can track all children at least until age 10. After the age of 10, the sample gradually decreases. Table 1 shows each age’s sample size.

In our empirical application, we examine the profile of ADHD pharmacological treatments by age and by school grade and consider two different definitions of the outcome variable: (1) a dummy variable for ADHD prescription at age *a*, $ADHD_{a}$, that takes value 1 if a child received at least one prescription related to ADHD in the period [a,a+1) where *a* is the child’s age in years and ranges from 5 to 15, and (2) a dummy variable for ADHD in school grade *g*, $ADHD_{g}$, that takes value 1 if a child received at least one prescription related to ADHD during the school grade *g* (September to August) with *g* starting from 0, the reception (kindergarten) year, and following with grades 1 to 10. In secondary analyses, we repeat the analysis using diagnoses defined analogously, rather than prescriptions for pharmacological treatment. To identify ADHD diagnoses, we use the Read Codes, a clinical terminology system used in general practices across the UK, and define ADHD using the same codes as Bushe et al. (2015).^23^ We define a child as diagnosed with ADHD at age *t* if either in his/her GP medical history there is at least one ADHD-related behavioral symptom recorded at any point in time up to age *t*, or if he/she has been prescribed ADHD-related medications at any point in time up to age *t* given that children cannot be prescribed without a formal diagnosis.^24^ This definition is relatively lenient, so we treat diagnoses as a secondary outcome and report them separately in Appendix B. Our key regressor is *Early*, a dummy variable that takes value 1 for children born in July-August and 0 if born in September-October.

**Table 1:** Number of observations by age

Age	Observations
5	96,698
6	96,698
7	96,698
8	96,698
9	96,698
10	80,805
11	66,434
12	52,599
13	39,290
14	27,446
15	17,384

**Table 2:** Summary statistics, by school starting age

	Full sample	Early starters	Late starters	Difference
Sex, in percentage points				
Male	51.1	50.9	51.3	−0.4
Female (%)	48.9	49.1	48.7	0.4
Ethnicity, in percentage points				
White	54.6	54.8	54.3	0.6*
Non-white	14.7	14.9	14.6	0.3
Unclassified	30.7	30.3	31.1	−0.9***
Deprivation index quintiles, in percentage points				
Q1 (least deprived)	28.4	27.8	28.9	−1.1***
Q2	24.9	25.0	24.8	0.1
Q3	20.2	20.2	20.2	0.0
Q4	16.1	16.3	15.8	0.5**
Q5 (most deprived)	10.4	10.7	10.2	0.5**
Maternal age at birth, in percentage points				
≤20	7.5	7.4	7.6	−0.2
21-34	74.4	74.3	74.6	−0.3
35-39	15.6	15.7	15.4	0.3
≥40	2.5	2.6	2.4	0.2**
Maternal age (continuous)				
Maternal age, continuous	29.1	29.2	29.1	0.1***
	(5.6)	(5.6)	(5.6)	(0.0)
ADHD rates, in percentage points				
Average ADHD diagnosis	0.8	1.0	0.6	0.4***
Average ADHD prescription	0.6	0.7	0.4	0.3***
Ever diagnosed with ADHD	2.2	2.7	1.7	0.9***
Ever prescribed ADHD drugs	1.4	1.7	1.1	0.6***
Anxiety rates, in percentage points				
Average anxiety diagnosis rate	1.0	1.1	0.9	0.2***
Average anxiety prescription rate	0.5	0.6	0.4	0.1***
Ever diagnosed with anxiety	4.7	5.1	4.4	0.7***
Ever prescribed anxiety drugs	1.5	1.7	1.4	0.3***
Child admissions while aged 5 to 15, in percentage points				
Any admission	32.7	32.6	32.8	−0.2
Gastroenteritis	0.7	0.7	0.6	0.1
Chest pain	0.4	0.4	0.4	0.0
Epilepsy	0.4	0.4	0.5	−0.1
Ear infections	1.7	1.7	1.7	0.1
Asthma	3.8	3.6	3.9	−0.3***
Flu	0.1	0.1	0.1	−0.0
Pneumonia	0.5	0.4	0.5	−0.1*
Conjunctivitis	0.1	0.1	0.1	−0.0
Maternal health and health behaviors while the child was				
aged 0 to 4, in percentage points unless otherwise indicated				
Any admission	68.2	68.2	68.3	−0.1
BMI (kg/m2)	26.3	26.3	26.2	0.1*
	(5.5)	(5.5)	(5.5)	(0.0)
Overweight (BMI > 25 kg/m$^2$)	50.9	51.4	50.5	0.9**
Missing info BMI	44.9	44.9	45.0	−0.1
Smoker	20.7	21.0	20.4	0.6*
Missing smoking	26.0	25.9	26.2	−0.3
Alcohol consumption >2 units/day	4.7	4.8	4.7	0.1
Missing alcohol consumption	62.8	62.9	62.7	0.2
*N*	96,698	49,157	47,541	96,698

Notes: For dummy variables, we report the average expressed in percentages for the full sample in column 1, for early starters in column 2, for late starters in column 4, and the difference in the average between early and late starters in column 4. For continuous variables, we report the mean and standard deviation. $$^{*}$$ p<0.10, $$^{**}$$ p<0.05, $$^{***}$$ p<0.01

### Descriptive statistics

The variables used in our analysis are summarized in column 1 of Table 2, where we report their averages using the full sample. As expected, approximately half of our sample consists of males, and the other half consists of females. Half of the children are white, and 15% are nonwhite. We do not have ethnicity information for about one-third of the individuals in the sample, which we code as a separate category labeled “unclassified.” Around half of the individuals in our sample live in postal codes with a deprivation index in the bottom two quintiles, representing the least deprived areas.^25^ Over 7% of the children in our sample have a mother aged 20 or less. In the last two rows, we report the rate (in percentage points) of ADHD diagnosis and pharmacological treatment averaged across the age range from 5 to 15.

**Fig. 1:**
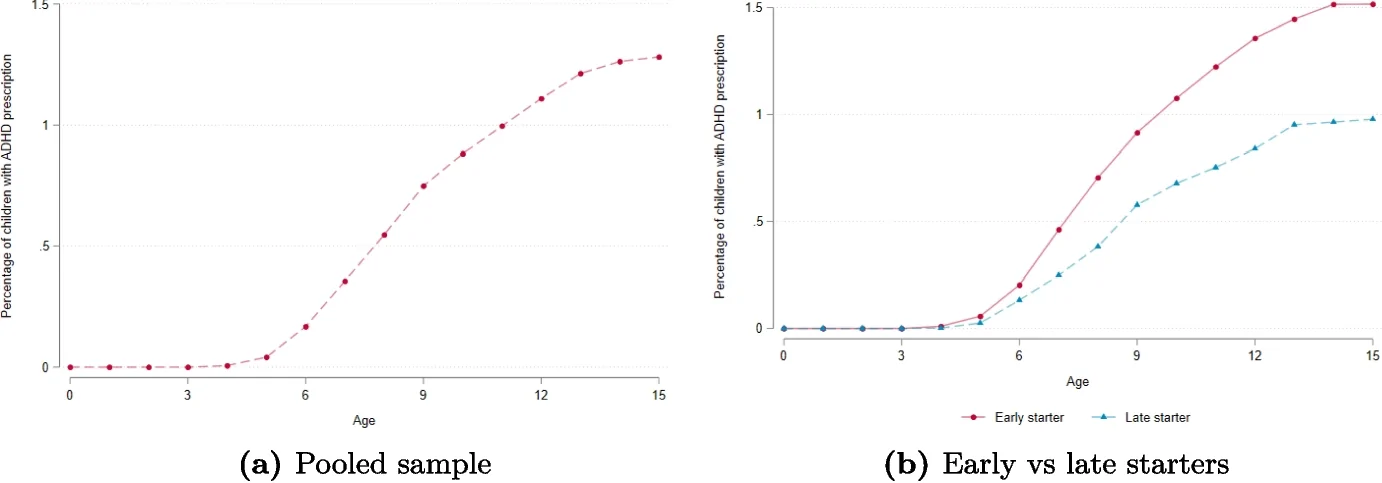
Rate of ADHD prescriptions by age. Notes: Each marker captures the percentage of children receiving an ADHD prescription in the corresponding age period. **a**) considers the full sample of children born between July and October, while **b**) considers early starters (born in July and August) and late starters (born in September and October) separately

In columns 2 and 3 of Table 2, we compare the characteristics of early school starters (children born between July 1 and August 31) relative to late starters (children born between September 1 and October 30). We find statistically significant differences between early and late starters for some of these characteristics. For example, early starters are less likely to live in the least deprived postal codes (bottom quintile) and more likely to live in postal codes in the top two quintiles. However, the only differences that are significant both statistically and in magnitude are the differences in the rate of diagnoses and prescriptions for ADHD. The rate of ADHD diagnosis increases by 50% for early starters relative to late starters, while the rate of drug prescriptions has an even larger increase of around 67%.

Next, we inspect the evolution of ADHD pharmacological treatment rates across ages 0 to 15 for the entire sample, considering the average of the dummy variable $ADHD_{a}$, which takes the value 1 if a child was prescribed at least one ADHD-related drug in the 1-year period [a,a+1). The average of $ADHD_{a}$ multiplied by 100 is the rate of prescription at age *a* expressed in percentage points, which we plot for each age from 0 to 15 in Fig. 1a. Thepercentage of children with an ADHD prescription is close to zero until the age of 5, which is in linewith the medical guidelines described in Section 3, and graduallyincreases to 1.25% by the time children are 15 years old. Figure 1b again plots theprofile of the ADHD prescription rate by age, but separately for early and late starters. As expected, after age 5, earlystarters are more likely to receive a prescription for ADHD, and the disparity between them and late startersgrows wider over time.^26^ A similar pattern holds for ADHD diagnoses(Appendix Fig. B.1).

While the main focus of our analysis is to explain the differences between early and late starters, for completeness, we also plot the ADHD pharmacological treatment rate by age for middle starters (that is, children born between November and June) in Appendix Fig. A.1. As expected, the prescription rates for middle starters are consistently below the prescription rates for early starters and above the rates for late starters.

## Empirical strategy

In England, most children start school full-time in September after their fourth birthday, by attending the reception year, which is equivalent to the US kindergarten year. Therefore, children born just before September start school almost 1 year earlier than children born on September 1 or soon after. In this section, we explain how we estimate the effect of being an early school starter (i.e., born in July–August) relative to late school starters (i.e., born in September–October) on the probability of receiving pharmacological treatment for ADHD.

Let us consider the dummy variable ADHD observed at a specific period in the child’s life *t*, which takes the value 1 if a child has received at least one prescription for ADHD in the 1-year period *t* and 0 otherwise. Because the rate of ADHD pharmacological treatment has been increasing across the years and varies across general medical practices, we control for the year of birth and general practice by estimating the following linear probability model:

6 $$\begin{aligned} ADHD_{i,t} = \alpha _t + \gamma _t \, Early_{i} + \beta _t \, X_{i} + \mu _{t,j} + \mu _{t,s} +\epsilon _{i,t}, \end{aligned}$$

where the subscripts *i*, *j*, *t*, and *s* denote, respectively, the child, the general medical practice where the child is registered, the time period in the child’s life when the ADHD prescription is observed,^27^ and the child’s year of birth (s=2002,...,2010). We observe children born between July and August, that is, within 2 months from the cutoff date for school entry, September 1. $Early_{i}$ takes the value 1 if the child *i* was born in July–August and 0 if born in September–October. $\mu _{t,j}$ and $\mu _{t,s}$ are the general practice and birth year fixed effects,^28^ respectively, and $\epsilon _{i,t}$ is the idiosyncratic error term. $\alpha _t$, $\gamma _t$, and $\beta _t$ are the intercept, the effect of being an early starter as defined in Eq. 2 in Section 2, and the vector of coefficients corresponding to the vector of background controls $X_i$. The vector of controls includes sex, ethnicity, the area deprivation index, and maternal age at birth (see Section 4 for descriptions of these variables). We account for heteroscedasticity by using robust standard errors using the Huber-White sandwich estimator.^29^

We estimate Eq. 6 separately for different values of *t* at which the child is observed. In our empirical application, we consider two ways to define the time *t*: age *a* and school grade *g*. To do so, we consider the following two outcome variables: (1) $ADHD_{i,a}$, that takes value 1 if a child received at least one prescription related to ADHD in the period [a,a+1) with a=5,...,15, and (2) $ADHD_{i,g}$, which takes the value 1 if child *i* received at least one prescription related to ADHD during the school grade *g* (September to August) with g=0,...,10.

$(\gamma _t \cdot 100)$ measures the effect of being an early starter as a percentage point increase in ADHD pharmacological treatment rate—the probability of receiving a pharmacological treatment for ADHD at time *t*. Because the rate of ADHD pharmacological treatment prescription varies substantially across age (see Fig. 1a), we present the effect of being an early starter in terms of percentage increase, rather than percentage points increase, in the ADHD pharmacological treatment rate at time *t*, i.e.,

7 $$\begin{aligned} r_t \cdot 100=\frac{Pr(ADHD_{i,t}=1|Early_i=1)-Pr(ADHD_{i,t}=1|Early_i=0)}{Pr(ADHD_{i,t}=1|Early_i=0)} \cdot 100, \end{aligned}$$

where the numerator can be replaced with $\gamma _t$ and the denominator with the rate of ADHD prescription for late starters at time *t*.

To consistently estimate the effect of being an early starter using model Eq. 6, the following conditions need to hold: (1) parents do not delay the start of the school of their children based on potential gains (i.e., no red-shirting), and (2) the month of birth is not related to unobserved child and family characteristics that may affect ADHD pharmacological treatment rates. Evidence on the validity of condition (1) has been provided by Crawford et al. (2013), who find that over 99% of children are enrolled in the correct academic year for their age, based on the full population of children in state (public) schools in England in 2008. If there was a substantial habit of red-shirting in our sample, our estimated effect could still be interpreted as a lower bound for the effect of early starters on the probability of receiving pharmacological treatment for ADHD. This is because red-shirting would lead parents to delay entry into the school, especially for more immature children, therefore attenuating the effect of being an early starter. The birth month can be correlated with child and family background characteristics (Buckles and Hungerman 2013; Shigeoka 2015). In our model, we address this endogeneity issue by controlling for general practice and year-of-birth fixed effects, along with a set of background characteristics.

Our rich administrative data on hospitals and general practices enable us to follow children over time and observe their ADHD prescriptions at each specific age between 5 and 15, thereby avoiding recall and measurement issues that typically affect parents’ and teachers’ reports on children’s ADHD in sample surveys. However, we do not observe the child’s age at school entry, so our estimate of the effect of starting school early should be interpreted as an intention-to-treat (ITT) estimate. We expect the ITT estimate to be close (if not identical) to the actual treatment effect in our sample, given that more than 99% of children are enrolled in the correct school grade based on their age (Crawford et al. 2013).

Because the baseline specification restricts attention to narrow birth-date windows around the school-entry cutoff and includes cohort and practice fixed effects, identification comes from local variation in date of birth rather than from long-run age trends. Formally, this specification can be viewed as similar to a regression discontinuity design with a 2-month bandwidth^30^:

8 $$\begin{aligned} \begin{aligned} ADHD_{i,t} = {}&\tilde{\alpha }_t- \tilde{\gamma }_t \, 1\!\!1(z_i \ge 0) +h_t^{-}(z_i)1\!\!1(z_i < 0)+h_t^{+}(z_i)1\!\!1(z_i \ge 0) \\&+ \tilde{\beta }_t \, X_{i} +\tilde{\mu }_{t,j} + \tilde{\mu }_{t,s} +\tilde{\epsilon }_{i,t}, \end{aligned} \end{aligned}$$

where the notation is the same as in Eq. 6, but we use the tilde sign to emphasize elements that may differ from the model Eq. 6. $z_i$ is the running variable, which is the birth date of child *i* centered around the cutoff date for school entry (September 1); $1\!\!1(z_i \ge 0)$ is a dummy taking value 1 if the running variable is positive (late school starters) and 0 otherwise. Notice that $\tilde{\gamma }_t$ captures the effect of being an early starter with respect to being a late starter when comparing individuals born just before and just after the cutoff point of September 1. To flexibly control for smooth relationships between the running variable and the outcome, we allow the functional form to differ on each side of the threshold by estimating separate local second-order polynomials, $h_t^{-}(z_i)$ and $h_t^{+}(z_i)$, for observations below and above the cutoff, respectively. We adopt a symmetric bandwidth of 60 days (2 months) on either side of the cutoff, ensuring identification relies on observations close to the threshold, where units are plausibly comparable.^31^ Notice that the discontinuity at the cutoff can be considered a sharp discontinuity, given that almost all children start school in September after they turn 4.

While it is, in general, important to specify the correct functions for $h_t^{-}(z_i)$ and $h_t^{+}(z_i)$ for the relationship between ADHD and the running variable, this specification is less crucial if we focus on a narrow bandwidth, i.e., a sub-sample of children born close to the cutoff point. The estimation of the model Eq. 6 is equivalent to the estimation of an RDD (regression discontinuity design) model with a bandwidth of 2 months on both sides of the cutoff date and where $h_t^{-}(z_i)$ and $h_t^{+}(z_i)$ are assumed to be the same ones, implying that the conditional expectation of ADHD varies only through a discontinuous shift at the cutoff rather than through smooth variation in the running variable. It is, therefore, unsurprising that the estimation of the model Eq. 6 produces results similar to the RDD model Eq. 8, as we show in Section 6.4.

## Results

In this section, we focus on differences in the prevalence of pharmacological treatment for ADHD between early and late starters. That is, we include children who have either a first-time (new) prescription or a renewed prescription initiated earlier.

**Fig. 2:**
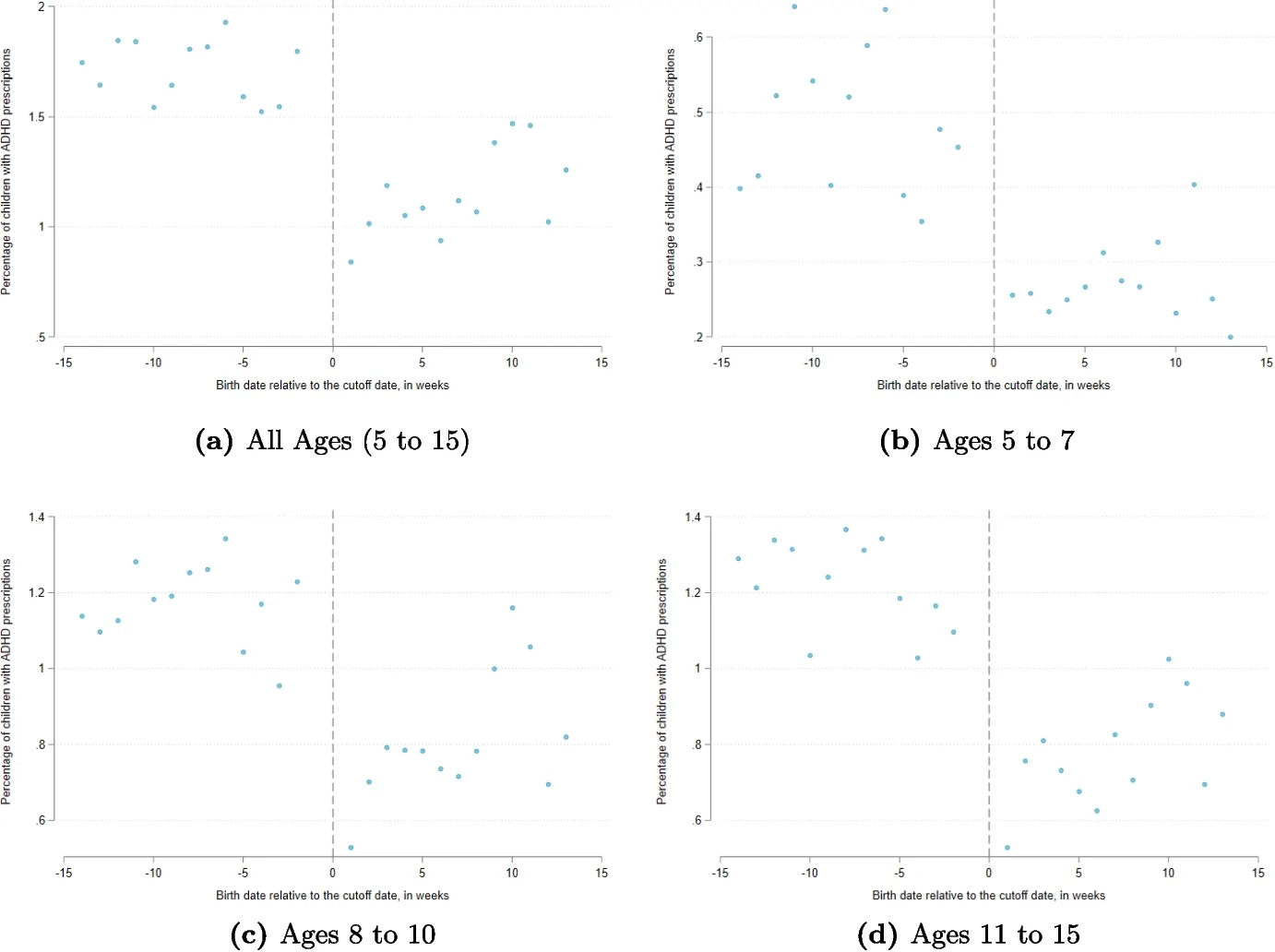
Prevalence of ADHD prescriptions by week of birth. Notes: Each marker captures the percentage of children receiving a prescription for ADHD by birth date in weeks relative to September 1. **a**) considers children at any age between 5 and 15, while **b**) to **d**) focus on different age groups: 5–7, 8–10, and 11–15. The vertical dashed line represents September 1, the cutoff point for school entry

### Descriptive evidence on our identifying variation

We compare ADHD pharmacological treatment rates between children born in July–August and those born in September-October (i.e., children born before and after the school entry cutoff for England). Figure 2a plots the ADHD pharmacological treatment rate against the week of birth, centered around September 1, for all children aged 5 to 15. In sub-figures b to d, we repeat this exercise separately for children aged 5–7, 8–10, and 11–15. All four sub-figures show a clear discontinuity in ADHD prescription rates at the school entry cutoff.

**Fig. 3:**
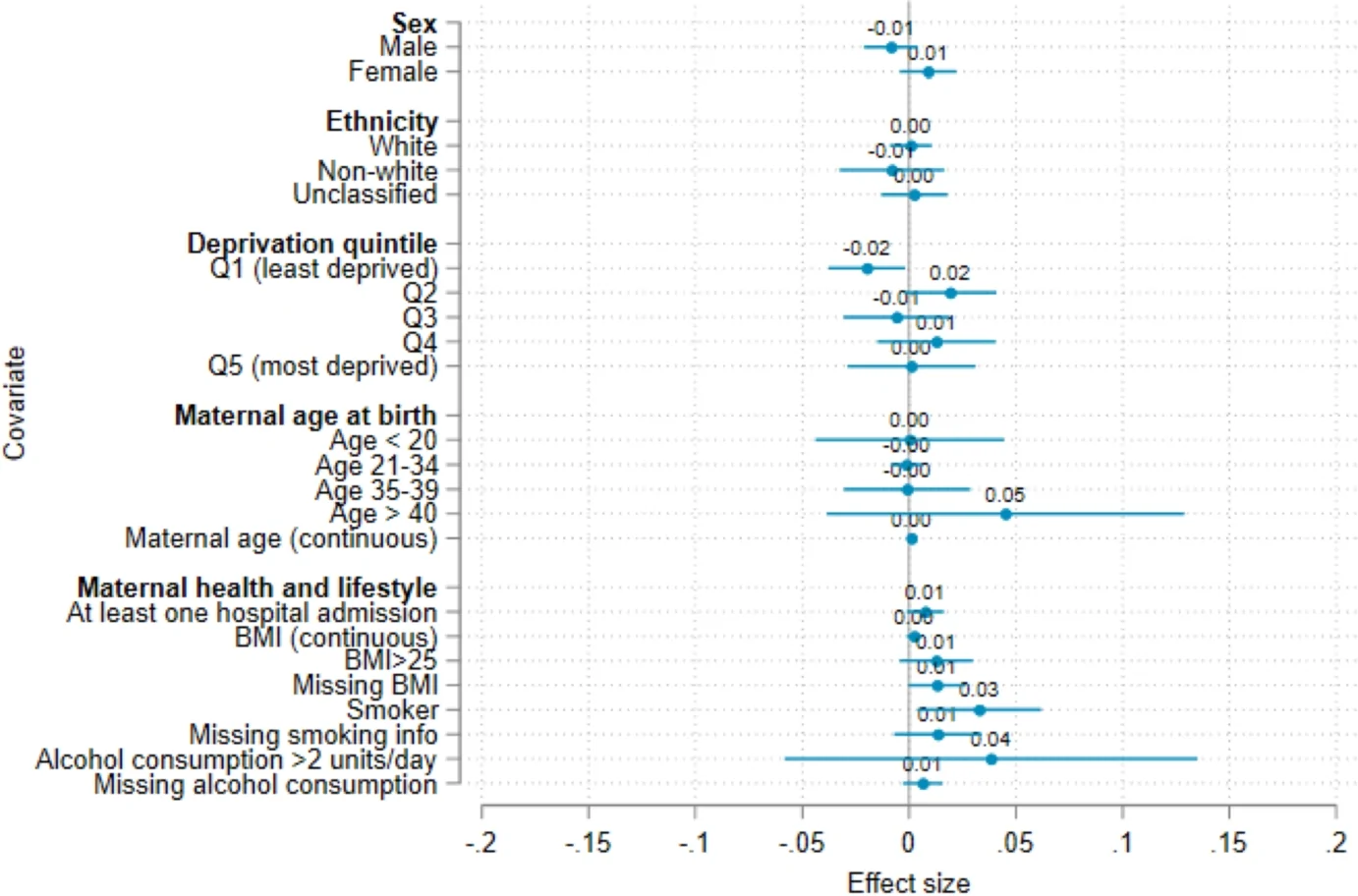
Balance of observable characteristics after controlling for general practice and year fixed effects. Notes: Each covariate is regressed on the dummy for early starters while controlling for general practice and year-of-birth fixed effects. This plots the estimated coefficient on the early-starter indicator and the corresponding 95% confidence intervals. Covariates include child gender, ethnicity, family socioeconomic status measured by Townsend deprivation quintiles, maternal age at birth, and a set of predetermined maternal health and lifestyle characteristics observed prior to school entry (i.e., when their child is between ages 0 and 4). Maternal health and lifestyle care include an indicator for at least one hospital admission while the child is aged 0–4 (from Hospital Episode Statistics), as well as maternal smoking status, alcohol consumption, and body mass index derived from primary care records and measured prior to school entry. Maternal BMI is defined as the average of all recorded BMI measurements over the same period, and an indicator for overweight status takes value 1 if this average exceeds 25. Maternal smoking is defined as an indicator equal to 1 if the mother is recorded as a light, moderate, or heavy smoker at least once, and 0 if records indicate only non-smoker or ex-smoker status. Alcohol consumption is defined as an indicator equal to 1 if there is evidence of average consumption of at least two daily units while the child is aged 0 to 4 in the primary care records, and 0 if and only if there is evidence of lower or no consumption. Because lifestyle variables have missing values, we also examine differences in the share of missing values

We investigate whether the sorting into July-August and September–October births is related to family background by comparing the average of background characteristics between early and late starters, while controlling for the year of birth and general practice fixed effects, as in our regression Eq. 6. Our results do not suggest any issue of endogeneity, as shown by the fact that the effect of the dummy *Early* on each of the control variables is never significantly different from zero at 5% level once we control for general practice and year-fixed effects (see Fig. 3).^32^ Even though including these background variables is not necessary for identification, we retain them in Eq. 6 to increase estimation precision.

To further assess balance around the school-entry cutoff, we extend the analysis in Fig. 3 to include a set of predetermined maternal health and lifestyle characteristics observed prior to school entry. Using linked hospital and primary care records, we test for discontinuities in maternal hospital admissions and health behaviors measured while the child is aged 0 to 4.^33^^,^^34^ We also examine maternal smoking status, alcohol consumption, and body mass index (BMI) using primary care records.^35^ For each of these variables, we estimate the effect of the early-starter indicator using the same specification as in Eq. 6, controlling for year of birth and general practice fixed effects. We find generally insignificant and small effects for these maternal characteristics. Because these variables are not observed for all mothers, we do not include them as baseline controls in our main specifications in order to preserve sample size and avoid selection driven by missing data.

**Table 3:** Effect of early start of school on ever being prescribed ADHD pharmacological treatment

	Ever prescribed ADHD drugs
	(1)
Early start	0.006$$^{***}$$
	(0.001)
N	96,698
Mean ever being prescribed for late starters, $Pr(EverADHD_{i,15}=1|Early_i=0)$	0.011
Effect size, $\gamma _{15}/Pr(EverADHD_{i,15}=1|Early_i=0)$	0.611

Notes: This reports the estimated $\gamma _{15}$ in Eq. 6 when the dependent variable is an indicator taking value 1 if the individual received at least one ADHD drug prescription up to age 15. The effect size is expressed as the proportional increase in the rate of having ever been treated with pharmacological treatment for early starters relative to late starters, $r_{15}=\gamma _g/Pr(EverADHD_{i,15}=1|Early_i=0)$. We control for sex, ethnicity, postal code SES, and maternal age at birth and include general practice and year of birth fixed effects

Finally, the regression discontinuity approach described in Eq. 8 assumes that children and families are randomly assigned to the treatment and control groups at the cutoff date for school entry. There should be no bunching of the child’s date of birth to the right or to the left of September 1. We evaluate the validity of this assumption by plotting the histogram of birth dates in days, with a 60-day bandwidth around the cutoff for school entry. Following recent developments in the literature on RDD, we also implement a nonparametric density estimator based on local polynomial techniques to evaluate the continuity of the density function around the cutoff date as proposed by Cattaneo et al. (2020). Appendix Fig. A.2 summarizes the results of this exercise. We cannot reject the null hypothesis of no discontinuity in the density function at the cutoff point (conventional *p*-value = 0.587, robust *p*-value = 0.588), suggesting no manipulation and supporting the assumption of random sorting at the cutoff.

Before examining the effect of school starting on ADHD pharmacological treatment by age and grade, we estimate its effect on the extensive margin of ADHD pharmacological treatment, i.e., on an indicator equal to 1 if a child received at least one prescription for ADHD by age 15. Table 3 shows that early starters are 61% more likely to receive at least one prescription.^36^ For completeness, we also show diagnoses: early starters are 53% more likely to have ever been diagnosed with ADHD (Appendix Table B.1).

### Gaps in prevalence rates of ADHD pharmacological treatment by age and school grade: exploring mechanisms

Recall from Section 2 that the effect of being an early starter when observing both early and late starters at the same age *a* is given by $\gamma _{a}= \gamma _{EXP,a} - \gamma _{AGEE,a} - \gamma _{RELAGE,a}$, while the effect measured for children in the same grade *g* is $\gamma _{g} = - \gamma _{AGEE,g} - \gamma _{RELAGE,g} - \gamma _{ABSAGE,g}$, and we compare the same school grade effect $\gamma _g$ with the same age effect $\gamma _a$ computed at age a=g+5. Recall also that we expect $\gamma _{EXP,t}>0$, $\gamma _{ABSAGE,t}>0$, $\gamma _{RELAGE,t}<0$ and $\gamma _{AGEE,t}<0$, which implies that $\gamma _{g}$ will provide an underestimation of the effect of being an early starter because biased by the absolute age effect, while $\gamma _{a}$ will provide a larger estimation which includes the effect of length of school exposure (grade). Appendix Table A.1 provides a summary of how these effects map into the coefficients of the model.

**Fig. 4:**
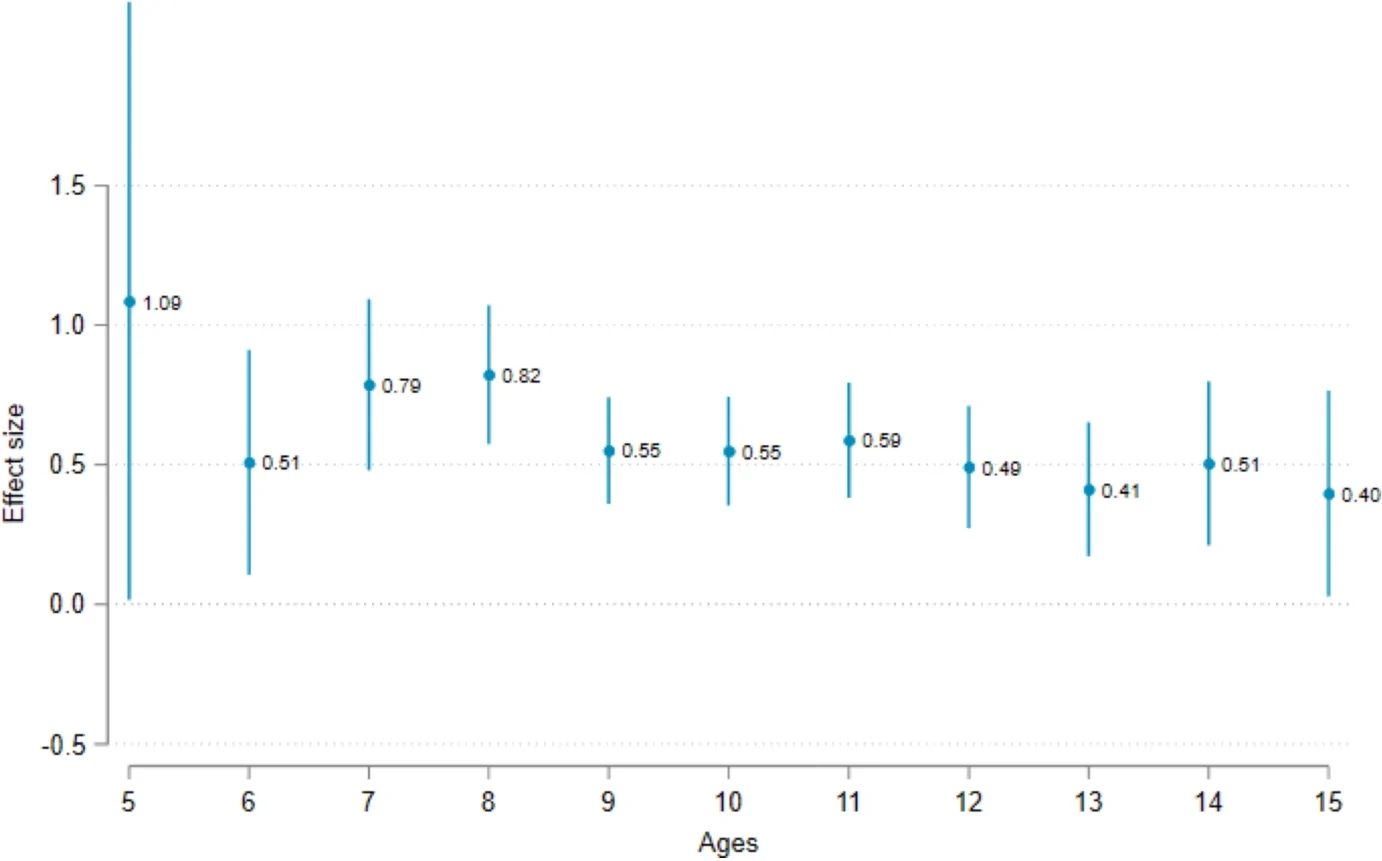
Effect of early start of school on ADHD prescriptions by age. Notes: This plots point estimates and 95% confidence intervals of separate regressions, one for each age from 5 to 15. The effects are expressed as a proportional increase in the prescription rate for early starters relative to late starters, $r_a=\gamma _a/Pr(ADHD_{i,a}=1|Early_i=0)$. In each regression, we control for sex, ethnicity, postal code SES, and maternal age at birth and include general practice and year of birth fixed effects

We begin by examining the effect of being an early starter on ADHD pharmacological treatment rates, comparing early and late starters at the same age *a*, $\gamma _{a}$. To do so, we estimate Eq. 6 with the dummy variable as the outcome, taking a value of one if a child was prescribed drugs related to ADHD in the interval [a,a+1), where age *a* ranges from 5 to 15. The results are presented in Fig. 4, where we plot the proportional increase in the ADHD prescription rate caused by being an early starter (the coefficient $r_a$ in Eq. 7) with the corresponding 95% confidence interval. Our findings indicate that early starters are more likely to receive an ADHD prescription at all ages, from 5 to 15. The percentage increase in the ADHD prescription rate for early starters relative to late starters is statistically significant at the 5% level at all ages and varies between 40% and 100%.^37^ The effect at age 15 is lower than in Table 3, partly because the sample is not fully tracked until age 15, and partly due to imperfect treatment adherence.

These results align with previous studies that have found that being an early starter leads to increased rates of ADHD pharmacological treatment, regardless of the estimation technique or cutoff date, which eliminates the seasonal effect as an explanation for these results. For instance, Elder (2010) finds that in the US, early starters are about 60% more likely to be diagnosed with ADHD and twice as likely to use ADHD-related stimulants regularly in grades 5 and 8. Similarly, Evans et al. (2010) find that, in the US, starting school earlier increases the ADHD prescription rate by about 25% relative to the mean. Recent work by Persson et al. (2025) confirms these findings in Sweden, where early starters are about 30% more likely to receive ADHD medications than late starters.

**Fig. 5:**
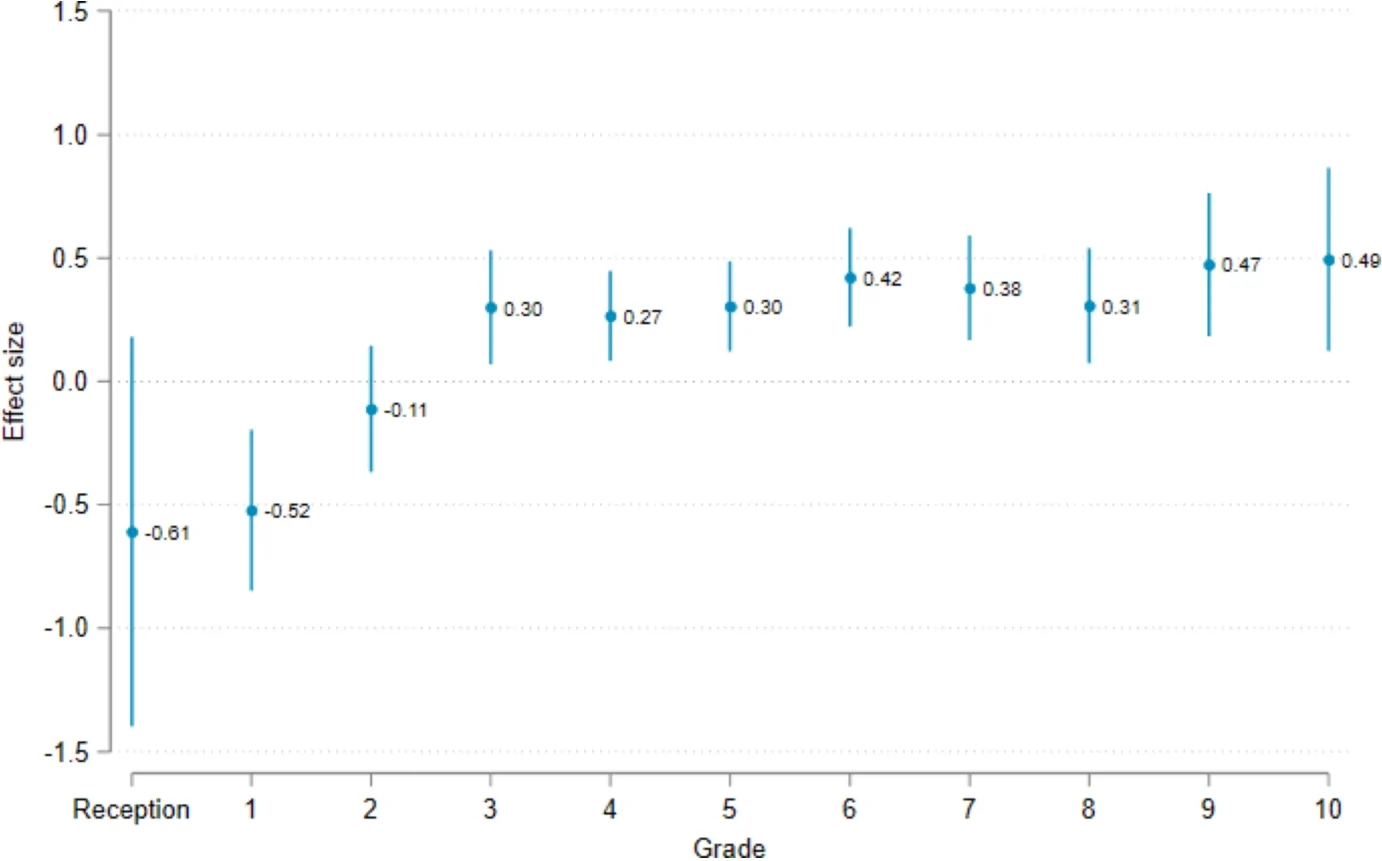
Effect of early start of school on ADHD prescriptions by grade. Notes: This plots point estimates and 95% confidence intervals of separate regressions, one for each grade from reception class to grade 10. The effects are expressed as a proportional increase in the prescription rate for early starters relative to late starters, $r_g=\gamma _g/Pr(ADHD_{i,g}=1|Early_i=0)$. In each regression, we control for sex, ethnicity, postal code SES, and maternal age at birth and include general practice and year of birth fixed effects

We then estimate $\gamma _g$, the effect of being an early starter within the same school grade. Figure 5 shows negative effects in reception and grades 1 and 2, and positive effects from grade 3 onward (30%–50%). The early negative estimates reflect the absolute age effect $\gamma _{ABSAGE,g}$, especially in reception and grade 1, when early starters are not yet 5. Appendix Fig. B.2 confirms this pattern using GP diagnoses, consistent with guidelines discouraging ADHD prescriptions before age 5. After age 7, the estimates based on diagnosis converge, and the early negative coefficients become statistically insignificant.

Few prior studies examine ADHD prescription rates by grade. Two exceptions, Pottegård et al. (2014) and Schwandt and Wuppermann (2016), show patterns in Denmark and Germany similar to ours: small or negative effects in the early grades, increasing through grades 3–4, then stabilizing.^38^

**Fig. 6:**
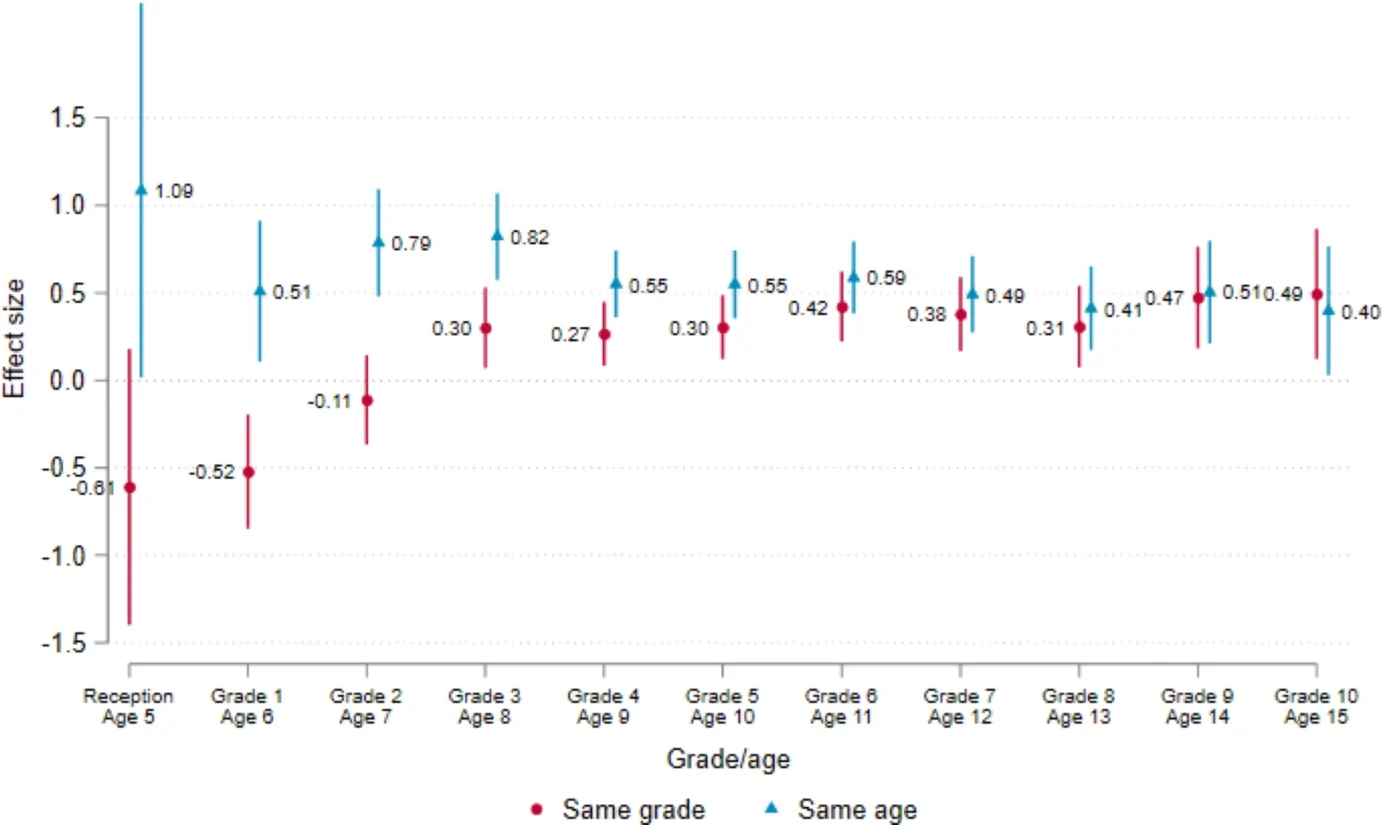
Effect of early start of school on ADHD prescriptions by age and grade. Notes: This plots point estimates and 95% confidence intervals of separate regressions, one for each age from 5 to 15 and grade from reception class to grade 10. The effects are expressed as a proportional increase in the prescription rate for early starters relative to late starters, $r_t=\gamma _t/Pr(ADHD_{i,t}=1|Early_i=0)$ with t=a for age and *g* for grade. In each regression, we control for sex, ethnicity, postal code SES, and maternal age at birth and include general practice and year of birth fixed effects

Finally, we compare $\gamma _a$ and $\gamma _g$ (the effects of being an early starter by age and grade) as shown in Fig. 6. The difference between them identifies the sum of the absolute age and the length of school exposure effects, $(\gamma _{ABSAGE,a} + \gamma _{EXP,g})$, per Eq. 5. This combined effect tends to zero from grade 3 onward.^39^

**Table 4:** Testing differences between same age and same grade estimates on the effect of early start of school on ADHD prescriptions

Age/grade	Same age	Same grade	*p*-value
	(1)	(2)	(3)
Age 5/reception	0.027	−0.014	0.058
	(0.014)	(0.009)	
	[1.086]	[−0.609]	
Age 6/Grade 1	0.067	−0.063	0.037
	(0.027)	(0.020)	
	[0.509]	[−0.522]	
Age 7/Grade 2	0.195	−0.027	0.036
	(0.039)	(0.032)	
	[0.787]	[−0.112]	
Age 8/Grade 3	0.315	0.109	0.052
	(0.049)	(0.043)	
	[0.823]	[0.301]	
Age 9/Grade 4	0.319	0.149	0.077
	(0.056)	(0.052)	
	[0.551]	[0.266]	
Age 10/Grade 5	0.373	0.206	0.097
	(0.067)	(0.063)	
	[0.549]	[0.304]	
Age 11/Grade 6	0.443	0.308	0.141
	(0.079)	(0.075)	
	[0.588]	[0.422]	
Age 12/Grade 7	0.414	0.316	0.226
	(0.094)	(0.090)	
	[0.492]	[0.379]	
Age 13/Grade 8	0.393	0.286	0.253
	(0.116)	(0.111)	
	[0.412]	[0.307]	
Age 14/Grade 9	0.488	0.448	0.602
	(0.144)	(0.140)	
	[0.505]	[0.473]	
Age 15/Grade 10	0.389	0.489	0.379
	(0.184)	(0.187)	
	[0.398]	[0.494]	

Notes: This reports the point estimates, standard errors (in parentheses), and effect sizes (in brackets) of separate regressions, one for each age from 5 to 15 (column 1) and grade from reception class to grade 10 (column 2). The estimates and standard errors are expressed in absolute values, while the effect sizes are expressed as a proportional increase in the prescription rate for early starters relative to late starters, $r_t=\gamma _t/Pr(ADHD_{i,t}=1|Early_i=0)$ with t=a for age and *g* for grade. These estimates are the same as the ones reported in Fig. 6. In each regression, we control for sex, ethnicity, postal code SES, and maternal age at birth, and include general practice and year of birth fixed effects. In column 3, we report the *p*-values for a test of the difference between the results in columns 1 and 2, assuming the null hypothesis of no difference between the estimates

To address concerns about the interpretation of relative effect sizes, we also report absolute differences (alongside the relative effect) and formally test for differences between the same-age and same-grade effects in Table 4. From age 8 (grade 3), these differences are no longer statistically significant, implying that we do not reject the assumption that $(\gamma _{ABSAGE,a}+\gamma _{EXP,g})=0$. The effect of absolute age $\gamma _{ABSAGE,a}$ is expected to be positive because the rate of ADHD prescription increases with age. We also expect the effect of the length of school exposure, $\gamma _{EXP,g}$, to be positive because early starters experience higher stress due to being in a higher grade. Under these expectations, $(\gamma _{ABSAGE,a}+\gamma _{EXP,g})=0$ implies that both $\gamma _{ABSAGE,a}$ and $\gamma _{EXP,g}$ tend to zero from age 8 (grade 3) onward and the effect of being an early starter ends up being driven by the effects of relative age and age at school entry.

**Fig. 7:**
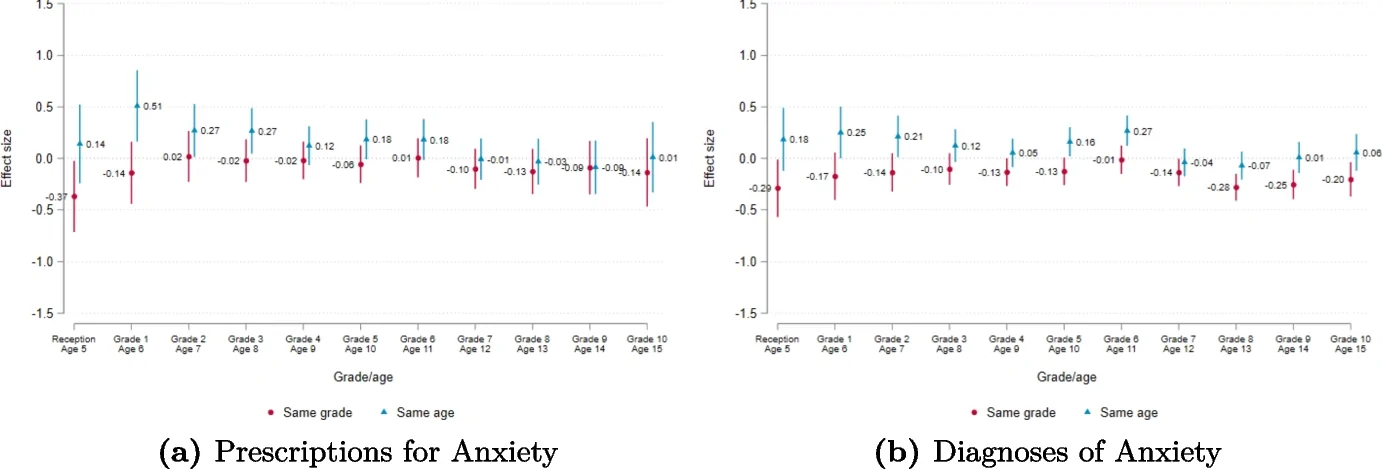
Effect of early start of school on anxiety prescriptions and diagnoses by age and grade. Notes: This plots point estimates and 95% confidence intervals of separate regressions, one for each age from 5 to 15 and grade from reception class to grade 10. The effects are expressed as a proportional increase in the prescription (**a**) and diagnosis (**b**) rate for early starters relative to late starters, $r_t=\gamma _t/Pr(Anxiety_{i,t}=1|Early_i=0)$ with t=a for age and *g* for grade. In each regression, we control for sex, ethnicity, postal code SES, and maternal age at birth and include general practice and year of birth fixed effects

Starting school early can cause stress for children, which may increase the likelihood of ADHD pharmacological treatment in the short term. While stress itself is difficult to quantify directly, we leverage data on anxiety disorder diagnoses and prescriptions for children in our sample. Anxiety disorders can be driven by remarkable manifestations of stress (Chaby et al. 2015; Rockhill et al. 2010).^40^ In Fig. 7, we estimate the impact of school starting age on both anxiety prescriptions (Fig. 7a) and diagnoses (Fig. 7b). For prescriptions, we observe smaller effects than those documented for pharmacological treatment of ADHD. Compared with Fig. 6, the estimates on anxiety are smaller and often statistically insignificant. Because prescriptions for anxiety may only capture extreme cases of stress, we also estimate the effects of starting school early on diagnoses of anxiety. While significant in some periods, these results are also closer to zero than our baseline results.^41^ While these findings provide suggestive evidence that anxiety is unlikely to be the main driver of our results, we emphasize that anxiety captures only one dimension of psychological distress. We therefore cannot exclude the presence of other context-induced symptoms or behaviors (observable to teachers and clinicians but unobserved in our data) that may differ by relative age.

Evidence from countries with school entry at age 6 also supports our conclusion. Even there, early starters show comparable increases in ADHD treatment.^42^ This suggests that uniformly raising school entry age by 1 year would not effectively reduce the ADHD treatment gap.

In sum, the relative age effect appears to be the main driver of the long-term ADHD treatment gap, leading to more marginal prescriptions for early starters. While children with severe symptoms are treated regardless of birth month, those with milder symptoms are more likely to be treated if they are early starters. This pattern suggests that school-entry timing can shift access to ADHD treatment at the margin. Whether this reflects overtreatment of early starters or undertreatment of late starters depends on the long-run benefits of marginal treatment, which we cannot observe directly.

Allowing parents to delay school entry for children born just before the cutoff may help reduce the ADHD treatment gap between children born on either side of the cutoff. However, it may worsen resource allocation if parents of children most in need of treatment delay both school entry and diagnosis. Alternatively, if high-SES parents are more likely to delay entry while low-SES children are more in need, red-shirting could shift resources from high- to low-SES groups, improving fairness. Yet even then, a gap would remain among low-SES children on either side of the cutoff, still pointing to inefficiency and inequity.

Since the long-term effects of being an early starter seem to be explained mainly by the relative age effect, a better solution than red-shirting could be sorting children into classes based on their months of birth to ensure that children have classmates of similar ages, or improving ADHD diagnosis by raising awareness of the relative age issues.

### Gaps in incidence rates of prescriptions: when do they initiate?

Figure 6 shows that the effect of being an early starter persists across ages. Why does this effect not diminish over time? One explanation is that ADHD is a chronic condition that often requires long-term pharmacological treatment. To examine this, we assess treatment persistence between ages 9 and 15 among children who received at least one prescription between ages 5 and 8. We find that 86.16% of children are still prescribed drugs for ADHD at age 9, 83.98% at 10, 81.29% at 11, 82.75% at 12, 74.63% at 13, 70.21% at 14, and 64.20% at 15 (Appendix Fig. A.4a). Moreover, this treatment persistence is present among both early and late starters (Appendix Fig. A.4b). Given this persistence, distinguishing between incidence and prevalence is important. Incidence refers to first-time prescriptions, while prevalence includes both initial and renewed treatments.^43^

**Fig. 8:**
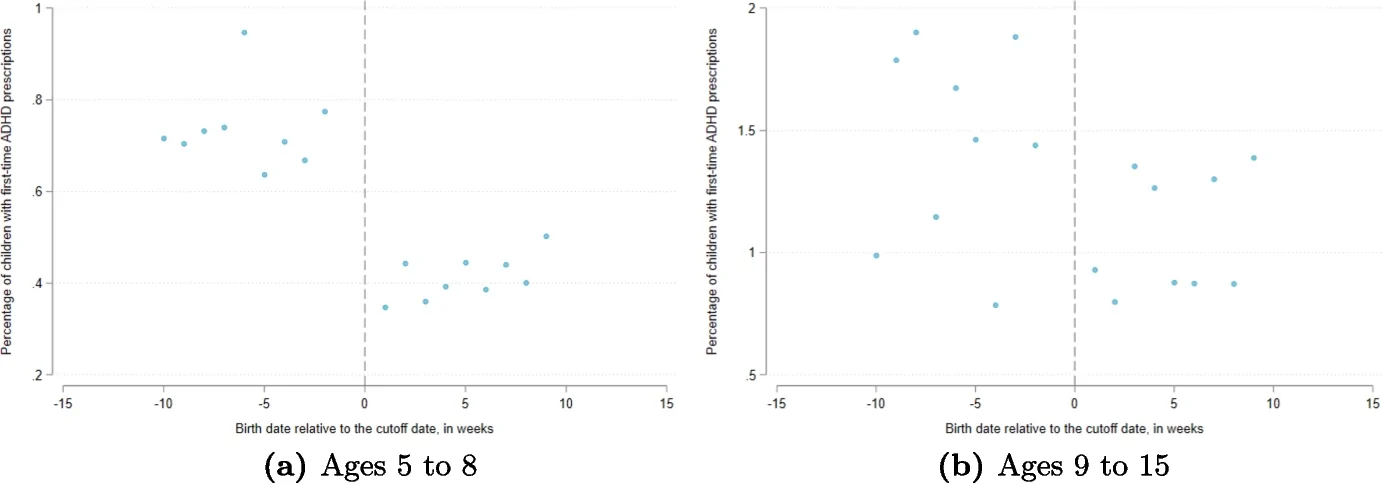
Incidence of ADHD prescriptions by week of birth. Notes: Each marker captures the percentage of children receiving a first-time prescription for ADHD by birth date in weeks relative to September 1. **a**) and **b**) consider prescriptions for children aged 5–8 and 9–15, respectively. The vertical dashed line represents September 1, the cutoff point for school entry

Plotting incidence rates against week of birth (centered on September 1), we find a clear discontinuity from age 5 to 8, but none after age 9 (Fig. 8). This suggests that the prevalence gap is driven entirely by prescriptions initiated before age 9.

**Fig. 9:**
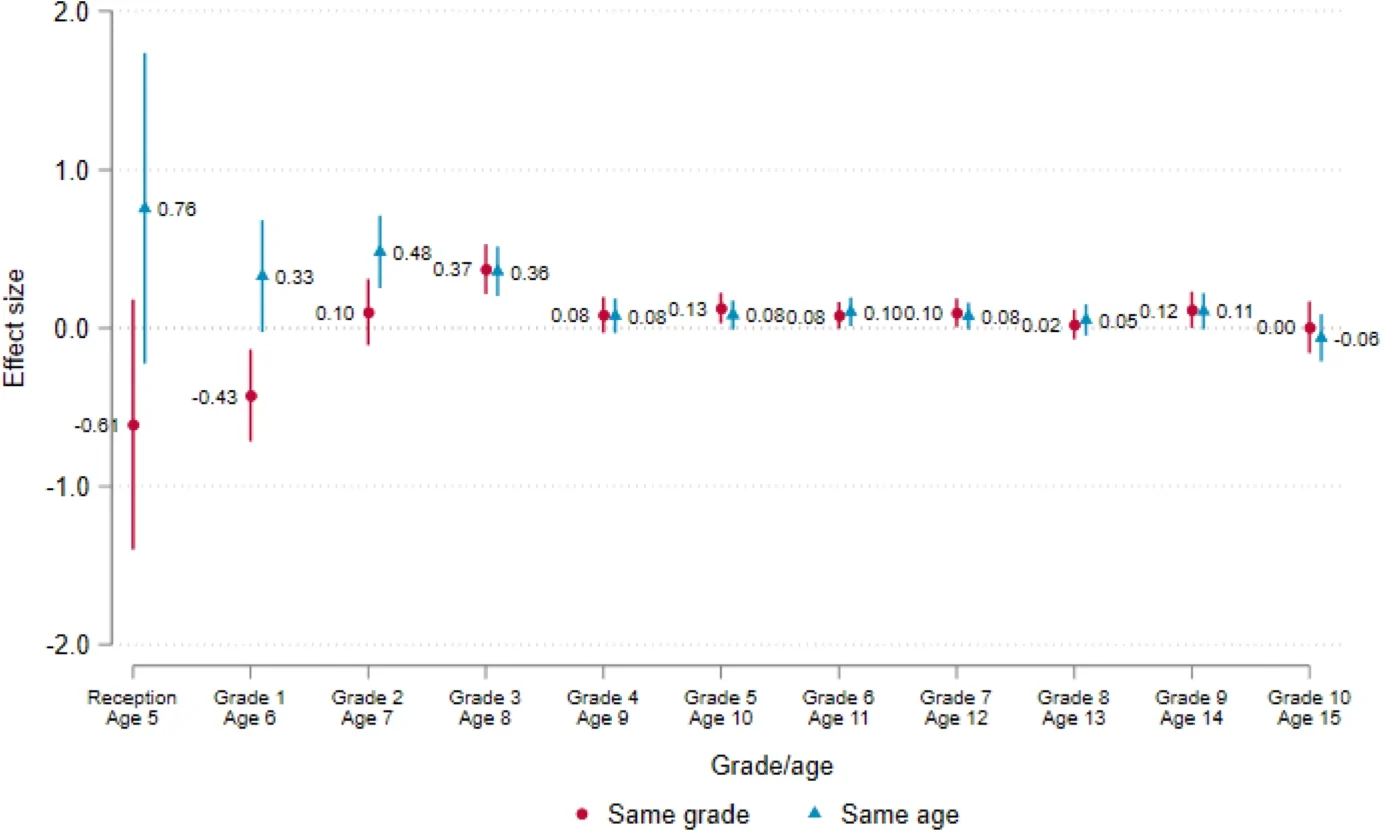
Effect of early start of school on ADHD first-time prescriptions by age and grade. Notes: This plots point estimates and 95% confidence intervals of separate regressions, one for each age from 5 to 15 and grade from reception class to grade 10. The effects measure the part of the proportional increase in ADHD pharmacological treatments explained by differences in first-time prescriptions between early and late starters (see Eq. 9). In each regression, we control for sex, ethnicity, postal code SES, and maternal age at birth and include general practice and year of birth fixed effects

We re-estimate Eq. 6 using a new dependent variable, $FirstPresc_{i,t}$, equal to 1 if child *i* receives their first ADHD prescription in year *t*, and 0 otherwise. The results are reported in Fig. 9 and expressed as the difference in the probability of first-time prescriptions in period *t* (either age *a* or grade *g*) between early and late starters over the probability of any prescriptions in period *t* for late starters,

9 $$\begin{aligned} pr_t=\frac{Pr(FirstPresc_{i,t}=1|Early_i=1)-Pr(FirstPresc_{i,t}=1|Early_i=0)}{Pr(ADHD_{i,t}=1|Early_i=0)}, \end{aligned}$$

where $ADHD_{i,t}$ in the denominator takes value 1 if a child receives either a new (first) or a renewed old prescription in period *t*.^44^

From age 9 (grade 4) onward, there is no effect of being an early starter on first prescriptions for ADHD, whereas there are effects up to age 8 (grade 3) similar in magnitude to the effects found in Fig. 6. Differences in ADHD drug prescriptions between early and late starters at later ages are explained by the persistence of treatment for ADHD initiated before age 9. Given the persistence in prescriptions over time, it becomes clear that an adequate diagnosis in the early years is essential to reduce the recurrent unfair difference in marginal treatments for ADHD between early starters and late starters. We find a similar pattern when considering diagnoses instead of drug prescriptions (Appendix Fig. B.3).^45^

Our finding of no effect on first prescriptions after age 8 aligns with Dalsgaard et al. (2012) and Dalsgaard et al. (2014a), who report little or no effect after age 7 in Denmark. This reinforces the need to address diagnostic biases in the early years of primary school.

### Robustness checks

**Fig. 10:**
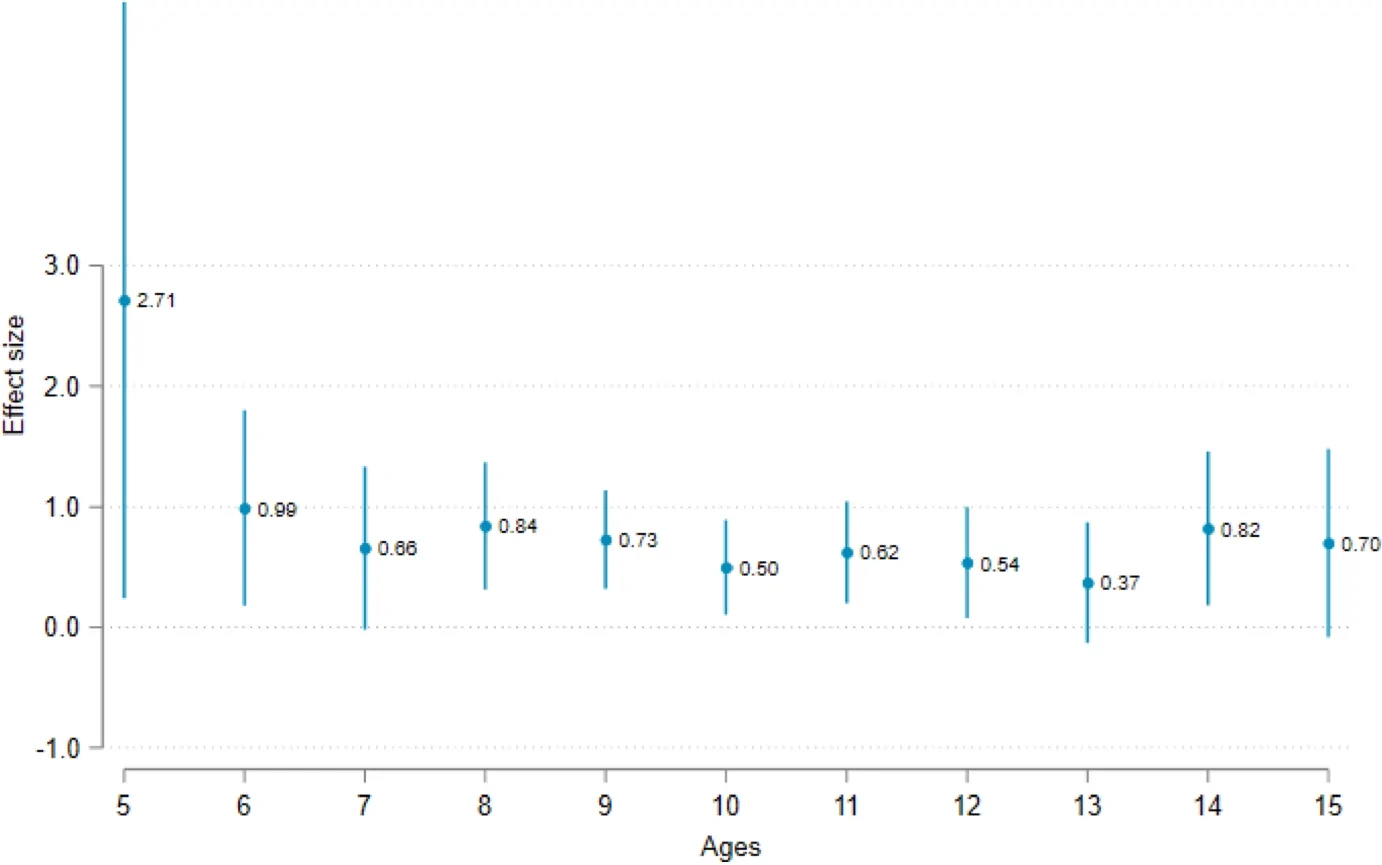
Effect of early start of school on ADHD prescriptions by age, using a regression discontinuity design. Notes: This plots point estimates and 95% confidence intervals of separate regressions, one for each age from 5 to 15. The effects are expressed as a proportional increase in the prescription rate for early starters relative to late starters, $r_a=\tilde{\gamma }_a/Pr(ADHD_{i,a}=1|Early_i=0)$. In each regression, we control for sex, ethnicity, postal code SES, and maternal age at birth and include general practice and year of birth fixed effects

We conduct several tests to evaluate the internal validity of our results. First, we address concerns about differences in unobserved characteristics between early and late starters using the RDD approach described in Section 5. Being born just before or after the cutoff date for school entry is more plausibly exogenous than being born in July–August rather than in September–October, so differences in unobservable characteristics are less of a concern when comparing individuals born around the cutoff date using the RDD.^46^ We report the effect of being born just before the cutoff date using the RDD approach at each age between 5 and 15 in Fig. 10. The effects are expressed as a proportional increase in ADHD prescription rate for children born immediately before compared to those born right after the cutoff date, as we do in our baseline results. Our estimates are similar to those reported in Fig. 4. This similarity reflects the fact that both the baseline and the RDD specifications rely on the same local variation in date of birth around the school-entry cutoff, rather than on extrapolation of age trends.

Second, we test the sensitivity of our results to using family fixed effects instead of general practice fixed effects. Following Dhuey et al. (2019); Chen et al. (2015), we compare siblings who just met or missed the cutoff for school entry. General practice fixed effects absorb time-invariant differences across practices and local areas, while family fixed effects absorb all time-invariant family background characteristics shared by siblings. The results are presented in Appendix Fig. A.6. As this approach constrains the sample, we expand the bandwidth from ±60 (Appendix Fig. A.6a) to ±120 days (Appendix Fig. A.6b). While the estimates are imprecise, they are qualitatively similar to our baseline results, particularly when using the larger bandwidth, consistent with the interpretation that unobserved family background characteristics are unlikely to be driving our results.

Third, we address concerns about measurement error in the date of birth in Appendix Fig. A.7. As detailed in Section 4, we do not observe exact birth dates; instead, we observe only the start and end of maternity care. For 99% of the sample, this span is 3 days or less, with an average and median around 2 days. We use the midpoint as a proxy for date of birth. Results remain virtually unchanged when we exclude children born within ±3 days of the cutoff. In addition, we re-estimate the main specifications using the beginning and the end of the maternity care spell as alternative definitions of date of birth. The estimated effects are very similar across all three definitions and our baseline specification.

Fourth, we estimate effects on a vector of placebo outcomes that should not be affected by relative age at school entry or by teachers’ assessments. Using Hospital Episode Statistics (HES) data on inpatient admissions, we construct binary indicators equal to one if a child experiences at least one hospital admission between ages 5 and 15 for a set of common pediatric conditions.^47^ Appendix Table A.5 shows generally statistically insignificant and economically small effects: only one outcome is statistically significant, and the estimated magnitudes are substantially smaller than those observed for ADHD treatment. Taken together, these results suggest that the early-late discontinuities documented for ADHD are unlikely to reflect broad differences in physical health or hospital utilization and instead are specific to outcomes related to ADHD diagnosis and treatment.

Fifth, we adopt a logistic regression rather than a linear probability model. We report the estimated odds ratios in Appendix Fig. A.8, i.e., the ratio of the odds of receiving a prescription for early starters to those for late starters at each age between 5 and 15. All odds ratios are statistically significantly greater than 1 and align with our baseline results.

Sixth, we examine whether our results are sensitive to attrition due to age-related right-censoring. In our data, attrition is mechanical and reflects the finite observation window rather than selective dropout. As a result, it does not affect age-specific estimates at younger ages, but it can influence cumulative outcomes such as whether a child is ever prescribed ADHD medication, which mechanically depends on the length of exposure. To address this concern, we re-estimate the effect of being an early starter on ever being prescribed pharmacological treatment for ADHD using weights proportional to each child’s observed length of exposure (Appendix Table A.6). The weighted estimates are very similar to the baseline results (see Table 3), suggesting that age-based right-censoring is unlikely to drive our findings.

We then examine whether the estimated effects differ across birth cohorts. This comparison is informative because, as discussed in Section 3, school entry rules may have changed over time for a subset of schools. While cohort fixed effects account for trends in prescriptions and school entry rules that affect early and late starters equally, cohort-specific changes could still bias our estimates if they generate differential prescribing trends between early and late starters. As shown in Appendix Fig. A.9, prescription rates have increased over time, consistent with trends observed in other developed countries such as the United States (Setlik et al. 2009). Nevertheless, when examining the effect of early school entry by age across three adjacent cohorts (2002–2004, 2005–2007, and 2008–2010), we find no statistically significant differences in the estimated effects (Appendix Fig. A.10). Together, these results suggest that neither right-censoring by age nor cohort-specific changes in school entry rules are likely to bias our estimates.

Finally, we examine whether the effect of early school entry varies systematically across general practice (GP) providers, which provides an indirect test of whether the estimated early-entry effect is driven by provider-specific diagnostic behavior. Because the QResearch data are not linked to school identifiers, we cannot include school fixed effects or school-by-early-entry interactions. Instead, we estimate specifications that interact the early-entry indicator with GP practice fixed effects, restricting the sample to practices with at least 200, 100, and 50 observations, respectively (Appendix Table A.7). Reporting the full set of interaction coefficients is impractical, so we summarize these results by computing the average marginal effect of early entry and comparing it to the baseline estimate. The marginal effects are very similar to those obtained from the baseline specification. Most importantly, if the early-entry effect were driven by GP-specific diagnostic bias, one would expect substantial heterogeneity in the GP-by-early-entry interaction coefficients. We formally test this and cannot reject the null hypothesis that these interaction effects are homogeneous across practices. Taken together, these results suggest that the early-entry effect is not driven by a small subset of providers with atypical diagnostic behavior, but instead reflects a systematic mechanism operating across practices.

## Interpretation of the gap in ADHD pharmacological treatment between early and late starters

**Fig. 11:**
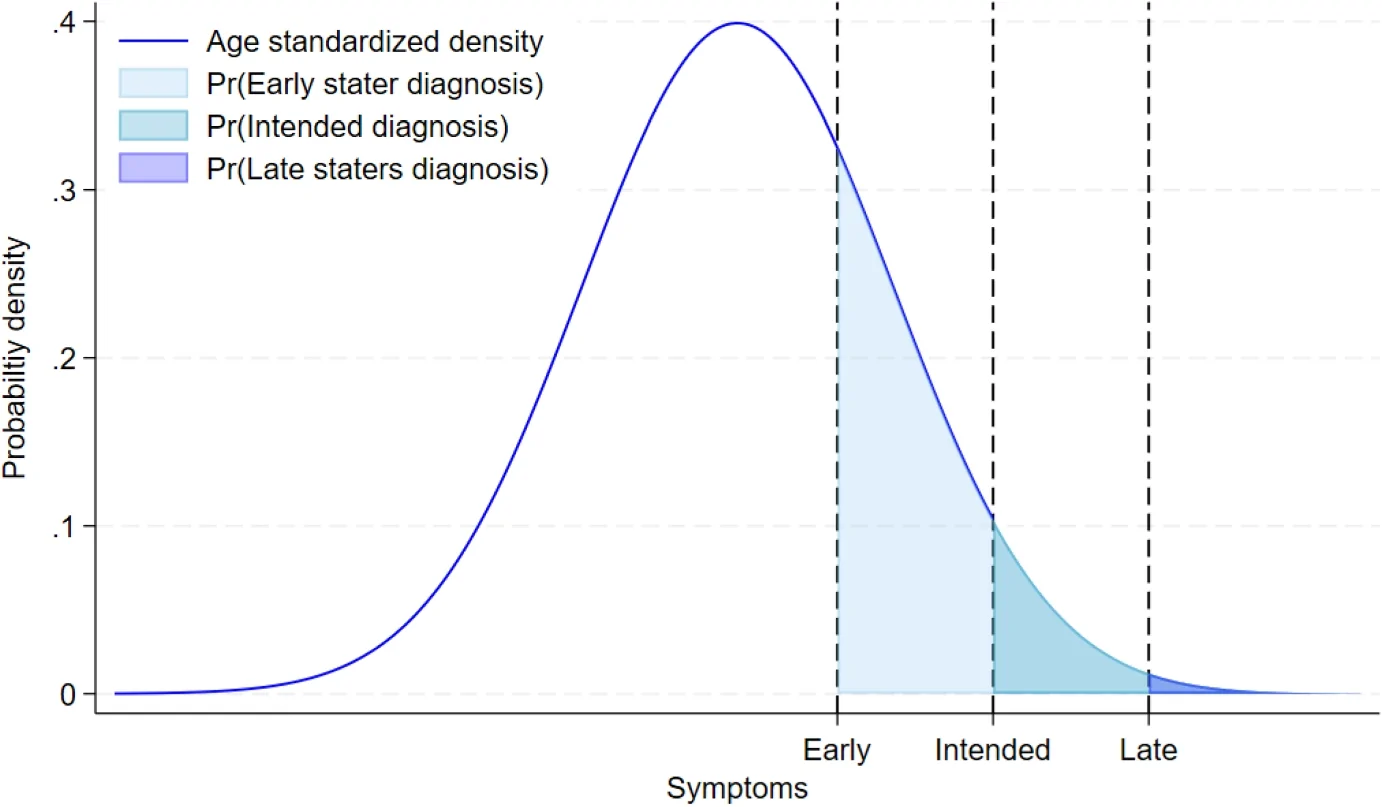
Stylized representation of the distribution of age-standardized ADHD symptoms with diagnostic thresholds. Notes: The vertical dashed lines represent the early starters, the intended (age-standardized), and late starters diagnostic thresholds potentially adopted by specialists

Every child can exhibit varying degrees of ADHD symptoms, including impulsiveness and inattention, which follow a natural distribution in the population. Diagnoses and treatment target those in the upper tail of this distribution. The observed gap in ADHD prescriptions between early (July–August) and late (September–October) starters suggests that diagnostic and treatment thresholds differ between the groups. This interpretation aligns with Persson et al. (2025) and is illustrated in Fig. 11. While children with pronounced symptoms are treated regardless of birth month, those with milder symptoms are more likely to be diagnosed and treated if born in July–August than in September–October.

**Fig. 12:**
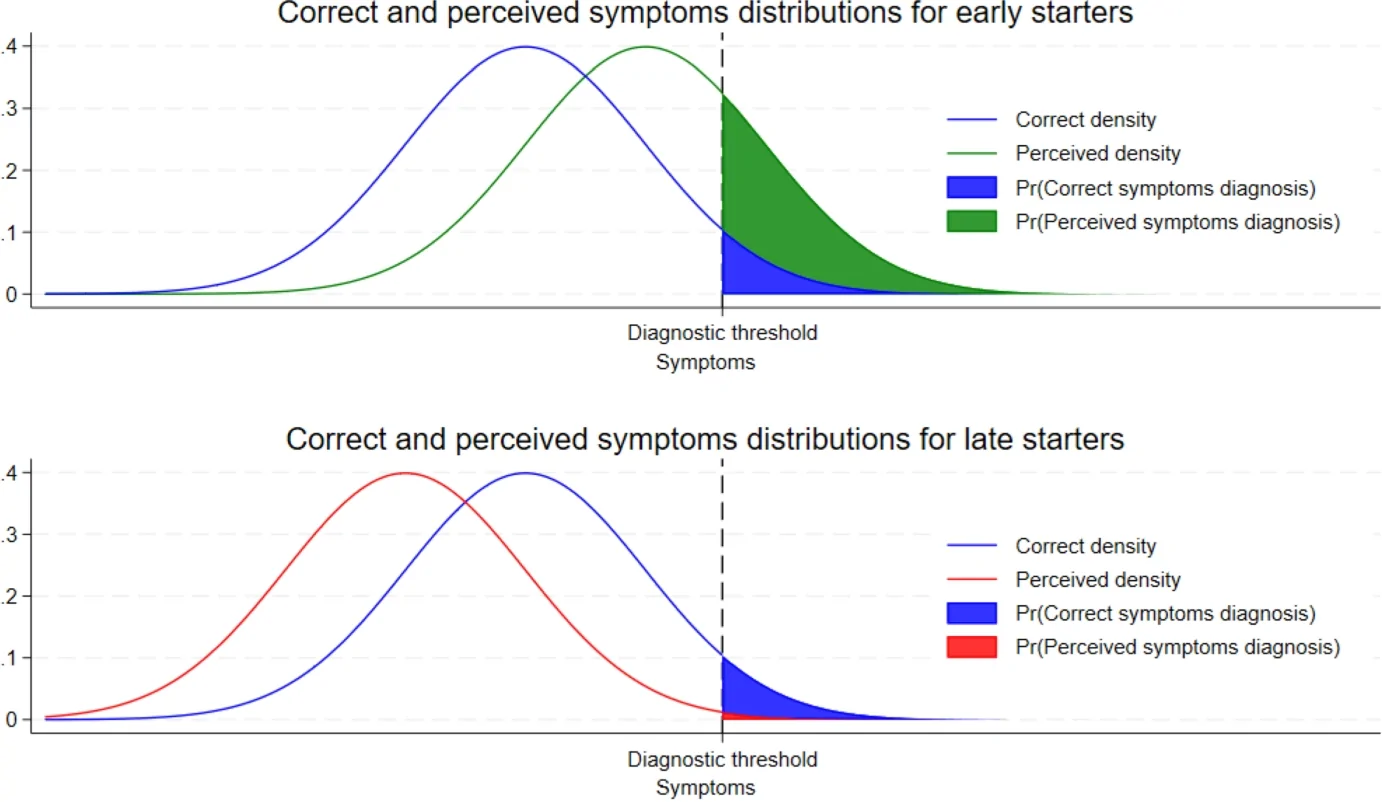
Stylized representation of the distributions of perceived and age-standardized (correct) ADHD symptoms for early and late starters. Notes: The vertical dashed lines represent the diagnostic thresholds teachers use

Why do thresholds differ between early and late starters? A common explanation is that teachers may perceive relatively younger children’s behavior as more severe in the classroom, where behavior is evaluated relative to peers and classroom expectations. Both Elder (2010) and Furzer et al. (2022) find that teacher-reported symptoms show a discontinuity at the school-entry cutoff, while parent-reported symptoms do not. This suggests that teacher perceptions (unadjusted for age) shift the symptom distribution rightward for early starters and leftward for late starters, as stylized in Fig. 12. The blue density reflects the age-standardized distribution, while the green and red curves represent unadjusted perceptions. Using non-age-standardized distributions inflates perceived symptom severity in early starters. Although clinical guidelines acknowledge age differences,^48^ diagnostic decisions rely on teacher reports, which can lead to lower effective thresholds for early starters and higher thresholds for late starters. This dynamic can contribute to marginal diagnoses and increased treatment among early starters. Although diagnostic criteria are formally defined by clinical guidelines, evidence suggests that behavioral assessments in school settings are often calibrated relative to grade-level expectations rather than chronological age (Efron 2017). As a result, similar behaviors may cross the threshold for concern more readily for relatively younger children, because classroom norms and expectations define what is considered appropriate behavior at a given grade level (Singh 2008).

Importantly, this mechanism does not require that teachers determine diagnoses or that clinical guidelines are not followed. ADHD diagnosis is carried out by specialists and relies on information from multiple settings, including school and home. Because teacher reports are a key input into both referral and clinical assessment (Schwandt and Wuppermann 2016), differences in how behavior is evaluated in the classroom can translate into differences in diagnosis, even when formal criteria remain unchanged.

The lower threshold for July–August births may lead to overdiagnosis and overtreatment; conversely, the higher threshold for September–October births may result in underdiagnosis and undertreatment. Identifying whether over- or underdiagnosis dominates would require knowing the scientifically appropriate threshold (data that are not available to researchers, as noted by Persson et al. (2025)).

As in previous studies, we cannot definitively assess whether early starters are undertreated or late starters are overtreated. Nonetheless, the increased treatment rate for early starters suggests a role for marginal diagnoses.^49^ We contribute to this debate by examining subgroup variation. Prior research has documented systematic differences in ADHD diagnosis and treatment across groups, conditional on symptoms. In particular, girls have been shown to be at higher risk of underdiagnosis relative to boys (Faraone et al. 2003). By contrast, several studies suggest that children from higher socioeconomic backgrounds may be more likely to receive an ADHD diagnosis for comparable behaviors, reflecting differences in referral patterns, parental advocacy, or diagnostic practices (Elder 2010; Emma Degroote and Houtte 2022; Elder and Zhou 2021). In this context, relative age at school entry could plausibly interact with these group-specific detection and referral processes.

However, because we do not observe underlying symptom severity or a clinically appropriate treatment threshold, and because subgroup estimates are often imprecise, we do not use our heterogeneity results to infer the direction of over- or underdiagnosis within specific groups. We therefore treat the heterogeneity analysis in Appendix C as exploratory and descriptive, and interpret it as evidence that relative-age mechanisms may operate differently across groups in ways consistent with patterns emphasized in the prior literature.

Finally, while ADHD prescription rates increase across birth cohorts in our data, particularly at older ages, we find that the early-late school entry gap is driven by differences in first-time prescriptions at younger ages and does not vary systematically across cohorts. This pattern suggests that the relative age mechanism operating at school entry is stable over time and distinct from the forces driving aggregate increases in ADHD diagnoses. As such, our results speak to marginal diagnosis at school entry rather than to changes in underlying mental health trends.

Consistent with this interpretation, cross-country evidence shows that relative-age gradients in ADHD diagnosis and treatment vary across institutional settings and are weaker or absent in some contexts, such as Denmark (Dalsgaard et al. 2012, 2014b; Pottegård et al. 2014), suggesting that features of the diagnostic and referral process influence the magnitude of these effects.

## Discussion

Children who start school at a younger age are more likely to be diagnosed with and receive pharmacological treatment for ADHD. Given the high prevalence of such treatment and its long-term impact on educational and labor market outcomes, it is important to understand the mechanisms behind this association. In particular, we investigate (1) what explains the effect of early school entry on the likelihood of ADHD diagnosis and treatment and (2) why this effect persists with age, unlike the impact on cognitive and socio-emotional skills found in prior research (Elder and Lubotsky 2009; Robertson 2011; Crawford et al. 2013).

Understanding these mechanisms is key to designing interventions that improve children’s health and education. We summarize our findings in two points. First, the persistence of the ADHD treatment gap after age 8 is primarily due to relative age effects. Second, the long-term impact of early school entry is driven by prescriptions initiated between ages 5 and 8.

Our results align with earlier studies in two ways. First, we replicate the observed increase in ADHD treatment rates among early starters. Second, we reinforce the hypothesis advanced by Evans et al. (2010); Elder (2010), and Furzer et al. (2022) that relative age differences are the main long-term driver of the ADHD treatment gap.

This relative age effect is consistent with teacher peer comparisons in early school years shaping referrals and diagnostic decisions for ADHD treatment. As a result, treatment gaps may reflect marginal diagnoses at best (Persson et al. 2025), and over- or underdiagnoses at worst (Furzer et al. 2022). Overdiagnosis can negatively affect schooling outcomes (Currie et al. 2014); underdiagnosis may impair future outcomes (Chorniy and Kitashima 2016) and have spillover effects on peers (Aizer 2008) and siblings (Breining 2014; Persson et al. 2025). Marginal diagnoses may yield limited or even harmful health effects, with long-run economic costs (Persson et al. 2025). Taken together, the treatment gap suggests that relative age can shift treatment decisions for children near the margin, which may not always align with underlying need.

Based on these findings, we suggest key recommendations for parents and policymakers. First, caution is warranted when considering delayed school entry (red-shirting). Our results suggest that changes in school entry timing can interact with relative age and institutional evaluation processes in early primary school, potentially leading to unintended consequences for diagnosis and treatment patterns.

Second, raising awareness of ADHD symptoms and relative age effects, especially among parents and teachers, could reduce biases in early referrals and diagnoses. Improving teachers’ ability to identify ADHD symptoms through targeted training is one avenue: Furzer et al. (2022) show that trained teachers do not exhibit relative age bias in symptom reports. Another option is to modify diagnostic protocols to reduce reliance on teacher reports for very early or late starters with mild symptoms. Persson et al. (2025) offer a similar recommendation to address “hereditary tagging,” where family history drives marginal diagnoses even in children with only mild symptoms.

Finally, schools could group students by narrower age bands (e.g., month or quarter of birth) to reduce peer age gaps. This could be done by adjusting classroom composition or introducing multiple school entry points throughout the year.

## Supplementary Information

Below is the link to the electronic supplementary material.

Supplementary file 1 (pdf 955 KB)

## Data Availability

This study used non-public microdata from QResearch via a remote access facility and complies with the relevant data agreement. QResearch pre-viewed the findings before publication to ensure that privacy-sensitive, individual-specific information is not revealed. We acknowledge the contributions of EMIS practices participating in QResearch and EMIS Health, and of the Universities of Nottingham and Oxford, for their expertise in establishing, developing, or supporting the QResearch database. This project involves data derived from patient-level information collected by the NHS as part of the care and support of cancer patients. The hospital, cancer, and mortality data are collated and maintained, and quality is assured by the National Disease Registration Service, which is part of NHS England. Access to the data was facilitated by the NHS England Data Access Request service. NHS England bears no responsibility for the analysis or interpretation of the data. We are happy to share their code with any user gaining access to the data to replicate our findings.

## Footnotes

1 If marginal treatments for early starters prove beneficial, this could lead to an unjust undertreatment of late starters compared to their early counterparts. Conversely, if such treatments have negative effects, early starters could be unfairly overtreated relative to late starters. In either case, the gap signals resource misallocation.

2 See, e.g., Brewer et al. (2007); Alalouf et al. (2024); Cuddy and Currie (2020); Einav et al. (2020); Persson et al. (2025); Currie and Zwiers (2025) and Bos et al. (2023).

3 See Elder and Lubotsky (2009); Evans et al. (2010); Elder (2010); Layton et al. (2018) for the US, Morrow et al. (2012) and Furzer et al. (2022) for Canada, Zoëga et al. (2012) for Iceland, Root et al. (2019) and Fleming et al. (2022) for the UK, Krabbe et al. (2014) for the Netherlands, Schwandt and Wuppermann (2016) for Germany, Chen et al. (2016) for Taiwan, and Persson et al. (2025) for Sweden. A notable exception is the case of Denmark, where Dalsgaard et al. (2012) and Dalsgaard et al. (2014a) do not find strong evidence of increased rates of ADHD among early school starters, while Pottegård et al. (2014) find some modest evidence. A review of the relationship between school starting age and ADHD diagnosis and treatment is provided in Whitely et al. (2018) and Schnorrbusch et al. (2020).

4 See, e.g, Elder and Lubotsky (2009); Elder (2010); Evans et al. (2010); Morrow et al. (2012); Schwandt and Wuppermann (2016); Bertoni et al. (2023).

5 A systematic review by Frisira et al. (2025) summarizes the existing evidence on the relative age effect on ADHD diagnoses and prescriptions.

6 Studies in behavioral genetics show that a stressful environment has an important role in explaining ADHD symptoms (Livingstone et al. 2016; Björkenstam et al. 2018; Humphreys et al. 2019; Hartman et al. 2019. Focusing on adolescents with ADHD and using a qualitative approach, Öster et al. (2020) link stress and ADHD symptoms.

7 See Cornelissen and Dustmann (2019) and Crawford et al. (2014) for similar models applied to other cognitive and socio-emotional skills.

8 Similarly to us, Evans et al. (2010) provide suggestive evidence that stress is not a relevant mechanism by showing that there are no gaps in ADHD diagnosis between early and late starters when looking at childhood ailments that may be amplified by stress, such as respiratory allergies, hay fever, and frequent headaches.

9 Date of birth is proxied using administrative maternity care records, which report the start and end of the maternity care spell rather than the exact birth date. In the main analysis, we use the midpoint of this spell as a proxy for date of birth. The results are robust to alternative definitions that use the beginning or the end of the maternity care spell, as well as to excluding observations close to the September 1 cutoff (see Section 4 for more detail and Section 6.4 for internal validity checks.

10 If we instead measure differences on the extensive margin (i.e., whether a child receives an ADHD prescription at least once by age 15), the difference is 61%.

11 The cited studies motivate the plausibility of short-run adjustment or stress responses at institutional transitions rather than persistent effects. Rimm-Kaufman and Pianta (2000) describe school entry as a salient developmental transition with heterogeneous adjustment by maturity. Using physiological measures, Dettling et al. (1999) and Ahnert et al. (2004) document elevated cortisol responses among younger children during transitions into structured group-care environments. Gunnar and Quevedo (2007) review evidence that stress responses to novel and socially demanding environments depend on developmental readiness and are strongest around periods of transition. This focus on early or immediate transitions may explain why we do not find evidence of a lasting stress-at-entry channel at later ages.

12 This effect is likely to be strongest in the early school years, when transitions into more structured and evaluative environments are relatively abrupt, and children have had limited time to adapt to classroom routines.

13 Kiessling and Norris (2023) show that a worsening of the relative rank of children with respect to their schoolmates leads to a deterioration of mental health even after controlling for their actual skills.

14 This model is similar to the model adopted by Cornelissen and Dustmann (2019) but it is extended to include the relative age as an additional mechanism. Equation :1 is a conceptual decomposition rather than an estimating equation. Because the length of school exposure, age at school entry, relative age, and absolute age are mechanically related, their coefficients cannot be separately identified in a single regression. We therefore allow all coefficients to vary freely by period *t* and exploit comparisons that hold different subsets of mechanisms constant. This approach avoids reliance on shared time trends and instead leverages institutional timing differences between age-based and grade-based comparisons.

15 Children in England start schooling with a reception year, which is equivalent to the US kindergarten year.

16 Community pediatricians are child health doctors trained in hospital and community settings. They have experience working with children and their families with various conditions and needs (see https://www.nhft.nhs.uk/community-paediatrics/ for more information).

17 Using data from a period when there was variation across schools in the age at the start of reception class, Cornelissen and Dustmann (2019) find that starting reception a few months earlier has a positive effect on cognitive and non-cognitive skills, especially for disadvantaged children.

18 As our data does not allow us to identify or isolate children from the minority of schools with multiple entry points, we checked for any potential bias by comparing the effects of early school entry on ADHD pharmacological treatment rates across different cohorts of children born from 2002 to 2010. Our analysis shows that the effect of early school entry on rates of pharmacological treatment for ADHD remained consistent across these cohorts.

19 As of 2015, children in England must do one of the following three until age 18: stay in full-time education; start an apprenticeship or traineeship; or spend 20 h or more a week working or volunteering while in part-time education or training.

20 A commissioned report by Hippisley-Cox et al. (2005) found QResearch to closely match the national sample of practices’ age-sex distribution and geographical spread. General practices within the QResearch consortium exhibit a marginally larger size (i.e., more registered patients) than the average practice in England, a phenomenon likely indicative of the willingness of large clinical practices to collaborate and exchange clinical data. Nevertheless, the patient demographic profiles within these QResearch-affiliated practices closely parallel the broader national patient demographics. Furthermore, prediction algorithms based on QResearch data have been validated against data from the Clinical Practice Research Datalink (CPRD) (Hippisley-Cox et al. 2014) and the UK Office for National Statistics (Nafilyan et al. 2021).

21 ADHD pharmacological treatments in the database are coded using British National Formulary (BNF; a standard drug reference text in the UK) codes (see, e.g., Baker et al., 2017).

22 Each of these four variables is divided by the appropriate count of households or persons to obtain a percentage score. The unemployment and overcrowding percentages are then log-transformed to normalize the raw values, which are highly skewed. All four variables are then standardized using a *Z*-score. The Townsend deprivation index is the sum of these four scores.

23 See Bushe et al. (2015) for a complete list of codes.

24 In other words, we assume there is a diagnosis whenever there is a prescription. Hence, the rate of diagnoses in our data is always higher than the rate of prescriptions.

25 In later analyses, we label the bottom two quintiles as “high SES” and the top two quintiles as “low SES” (see Section 6).

26 Beau-Lejdstrom et al. (2016) estimate the annual prevalence rates of ADHDpharmacological treatment among children below age 16 to be around 51 per 10,000 children,which is comparable to the estimates we report in Table 2. Similarly, McKechnie et al. (2023) estimate that1.4 to 2.4% of children in the UK between ages 10 and 16 have a prescriptionfor ADHD. Studies using prescription data from primary care records are likely to underestimate the total prevalence of ADHD pharmacological treatment, as children may also receive medication via other providers.

27 As detailed in the following paragraph, the time period *t* is defined either by age or by school grade, and Eq. 6 is estimated separately under each definition.

28 Birth-cohort fixed effects absorb cohort-specific trends in ADHD diagnosis and treatment that are common to children born in the same period, such as changes in diagnostic guidelines, prescribing norms, or awareness over time. As a result, identification does not rely on secular trends across cohorts but on within-cohort comparisons of early and late starters.

29 In our robustness checks, we show that our results do not change when using a logistic regression specification rather than a linear probability model.

30 Although the running variable is calendar time (date of birth), our design differs from a “pure” Regression Discontinuity in Time (RDiT) setting (Hausman and Rapson 2018). In RDiT designs, a policy change at a single date affects the same units over time. By contrast, our design exploits a birth-date cutoff that assigns different individuals to different school-entry cohorts, so identification comes from cross-sectional comparisons of children born just before and just after the cutoff. For this reason, standard RD diagnostics, including manipulation tests and local polynomial estimation within narrow windows, are appropriate in this setting.

31 When using covariate-adjusted bandwidth selection and robust bias-corrected inference (see Calonico et al., 2017), we find that the optimal bandwidth is between 57 and 89 depending on the child’s age. The results remain similar to those produced with the 2-month bandwidth. This suggests that the 2-month bandwidth used to select our sample of children is close to the optimal choice.

32 The only exception we find is when looking at the probability of living in the most deprived postal codes, which has a difference between early and late starters that is statistically significant at 5% level but it is very small in magnitude—2 percentage points.

33 All maternal health and lifestyle variables are measured prior to school entry to avoid post-treatment or bad-control concerns.

34 Maternal hospital admissions are defined as an indicator equal to one if the mother experiences at least one hospital admission while the child is aged 0 to 4, using Hospital Episode Statistics.

35 Maternal BMI is defined as the average of all recorded BMI measurements over the same period, and an indicator for overweight status takes value one if this average exceeds 25. Maternal smoking is defined as an indicator equal to one if the mother is recorded as a light, moderate, or heavy smoker at least once, and zero if records indicate only non-smoker or ex-smoker status. Alcohol consumption is defined as an indicator equal to one if there is evidence of average consumption of at least two daily units while the child is aged 0 to 4 in the primary care records, and zero if and only if there is evidence of lower or no consumption. Because lifestyle variables have missing values, we also examine differences in the share of missing values.

36 Because our data cover children born between 2002 and 2010 and extend to the end of 2020, not all cohorts are observed through age 15. As a result, this cumulative outcome may be affected by right-censoring for younger cohorts. In Section 6.4, we assess the sensitivity of these results to attrition by comparing estimates across cohorts and by re-estimating cumulative outcomes using exposure-length weights. These checks show that attrition is unlikely to be a driver of our results.

37 The estimates are less precise at age 5 because the ADHD rate at this age is extremely low, as shown in Fig. 1

38 These grade gaps appear in Fig. 2 in Pottegård et al. (2014). They are not directly reported by Schwandt and Wuppermann (2016) but can be inferred from their Fig. 2.

39 Figure 6 reports $\gamma _a$ and $\gamma _g$ divided by the ADHD prescription probability for late starters at age a=g+5 and grade *g*. Appendix Fig. A.3 shows that these denominators are nearly identical.

40 The guidelines for pharmacological treatment of children with anxiety are reviewed in Patel et al. (2018).

41 In Appendix Tables A.2 and A.3, we evaluate the difference between the “same age” and “same grade” coefficients for anxiety prescriptions and diagnoses, analogously to what we do in Table 4.

42 See, e.g., Elder (2010) for the US and Persson et al. (2025) for Sweden.

43 Appendix Fig. A.5 shows that most children receive their first ADHD prescription by age 9.

44 Note that the proportional increase in prevalence rate defined in Eq. 7 is

$$\begin{aligned} {\begin{matrix} r_t & =\frac{Pr(ADHD_{i,t}=1|Early_i=1)-Pr(ADHD_{i,t}=1|Early_i=0)}{Pr(ADHD_{i,t}=1|Early_i=0)}\\ & =\frac{Pr(FirstPresc_{i,t}=1|Early_i=1)-Pr(FirstPresc_{i,t}=1|Early_i=0)}{Pr(ADHD_{i,t}=1|Early_i=0)} \\ & +\frac{Pr(RenewPresc_{i,t}=1|Early_i=1)-Pr(RenewPresc_{i,t}=1|Early_i=0)}{Pr(ADHD_{i,t}=1|Early_i=0)}, \end{matrix}} \end{aligned}$$

so $pr_t$ in Eq. 9 is the part of the proportional increase in prevalence rate explained by first prescriptions.

45 A child is defined as having a first-time diagnosis at age *t* (grade *g*) if symptoms are first recorded at that age or, if absent, if treatment begins then.

46 The manipulation test does not reject of absence of discontinuity in the birth date at the cutoff (see Section 6.1).

47 Placebo outcomes include malnutrition, gastroenteritis, chest pain, epilepsy, ear infections, asthma, flu, pneumonia, conjunctivitis, and an indicator for any hospital admission. These conditions are chosen because they are typically diagnosed and treated based on clinical symptoms, laboratory tests, or acute medical presentation, rather than through school-based behavioral observation or teacher referral, as reflected in standard diagnostic classifications and clinical guidelines (e.g., ICD-based coding and NICE guidelines for pediatric asthma and epilepsy).

48 In England, the National Institute for Health and Care Excellence advises that “ADHD should be considered in all age groups, with symptom criteria adjusted for age-appropriate changes in behaviour.”

49 A related debate in the literature concerns whether increases in observed ADHD diagnosis and treatment reflect misdiagnosis and marginal treatment or genuine deterioration in child mental health. Our analysis does not aim to explain aggregate trends over time, nor can it distinguish correct from incorrect diagnoses, since we do not observe underlying symptom severity or a scientifically appropriate treatment threshold. Rather, our findings are most naturally interpreted through a threshold-based lens: relative age at school entry shifts treatment decisions for children near a latent diagnostic threshold. This interpretation is consistent with recent evidence summarized by Nicoletti and Vidiella-Martin (2025), who emphasize the role of changing diagnostic thresholds and marginal diagnoses in explaining rising ADHD diagnosis rates, as well as with structural evidence showing that substantial diagnostic disparities can arise from decision-making under uncertainty rather than from differences in underlying symptom prevalence (Marquardt 2025).

## References

[CR1] Ahnert L, Gunnar MR, Lamb ME, Barthel M (2004) Transition to child care: associations with infant-mother attachment, infant negative emotion, and cortisol elevations. Child Dev 75(3):639–650 15144478 10.1111/j.1467-8624.2004.00698.x

[CR2] Aizer A (2008) Peer effects and human capital accumulation: the externalities of ADD. Technical report, National Bureau of Economic Research

[CR3] Alalouf M, Miller S, Wherry LR (2024) What difference does a diagnosis make? Evidence from marginal patients. Am J Health Econ 10(1):97–131

[CR4] Aveyard P, Gao M, Lindson N, Hartmann-Boyce J, Watkinson P, Young D, Coupland CA, San Tan P, Clift AK, Harrison D et al (2021) Association between pre-existing respiratory disease and its treatment, and severe COVID-19: a population cohort study. Lancet Respir Med 9(8):909–923 33812494 10.1016/S2213-2600(21)00095-3 PMC8016404

[CR5] Baker R, Kendrick D, Tata LJ, Orton E (2017) Association between maternal depression and anxiety episodes and rates of childhood injuries: a cohort study from England. Inj Prev 23(6):396–402 28232401 10.1136/injuryprev-2016-042294

[CR6] Beau-Lejdstrom R, Douglas I, Evans SJ, Smeeth L (2016) Latest trends in ADHD drug prescribing patterns in children in the UK: prevalence, incidence and persistence. BMJ Open 6(6) 10.1136/bmjopen-2015-010508 PMC4932306 27297009

[CR7] Bedard K, Dhuey E (2006) The persistence of early childhood maturity: international evidence of long-run age effects. Q J Econ 121(4):1437–1472

[CR8] Bertoni M, Marin-Lopez BA, Sanz-de Galdeano A (2023) Subjective gender-based patterns in ADHD diagnosis. Technical report, IZA Discussion Papers

[CR9] Björkenstam E, Björkenstam C, Jablonska B, Kosidou K (2018) Cumulative exposure to childhood adversity, and treated attention deficit/hyperactivity disorder: a cohort study of 543,650 adolescents and young adults in Sweden. Psychol Med 48(3):498–507 28738913 10.1017/S0033291717001933

[CR10] Black SE, Devereux PJ, Salvanes KG (2011) Too young to leave the nest? The effects of school starting age. Rev Econ Stat 93(2):455–467

[CR11] Blanden J, Del Bono E, McNally S, Rabe B (2016) Universal pre-school education: the case of public funding with private provision. Econ J 126(592):682–723

[CR12] Bos M, Hertzberg A, Liberman A (2023) The effects of diagnosing a young adult with a mental illness: evidence from randomly assigned doctors

[CR13] Breining SN (2014) The presence of ADHD: spillovers between siblings. Econ Lett 124(3):469–473

[CR14] Brewer NT, Salz T, Lillie SE (2007) Systematic review: the long-term effects of false-positive mammograms. Ann Intern Med 146(7):502–510 17404352 10.7326/0003-4819-146-7-200704030-00006

[CR15] Buckles KS, Hungerman DM (2013) Season of birth and later outcomes: old questions, new answers. Rev Econ Stat 95(3):711–724 24058211 10.1162/REST_a_00314 PMC3777829

[CR16] Bushe C, Wilson B, Televantou F, Belger M, Watson L (2015) Understanding the treatment of attention deficit hyperactivity disorder in newly diagnosed adult patients in general practice: a UK database study. Pragmatic and Observational Research 6:1 27774030 10.2147/POR.S74161 PMC5045022

[CR17] Calonico S, Cattaneo MD, Farrell MH, Titiunik R (2017) rdrobust: software for regression-discontinuity designs. Stand Genomic Sci 17(2):372–404

[CR18] Cascade E, Kalali AH, Wigal SB (2010) Real-world data on: attention deficit hyperactivity disorder medication side effects. Psychiatry (Edgmont) 7(4):13 PMC2877616 20508803

[CR19] Cattaneo MD, Jansson M, Ma X (2020) Simple local polynomial density estimators. J Am Stat Assoc 115(531):1449–1455

[CR20] Chaby L, Cavigelli S, Hirrlinger A, Caruso M, Braithwaite V (2015) Chronic unpredictable stress during adolescence causes long-term anxiety. Behav Brain Res 278:492–495 25448433 10.1016/j.bbr.2014.09.003

[CR21] Chen K, Fortin N, Phipps S (2015) Young in class: implications for inattentive/hyperactive behaviour of Canadian boys and girls. Canadian Journal of Economics/Revue canadienne d’économique 48(5):1601–1634

[CR22] Chen M-H, Lan W-H, Bai Y-M, Huang K-L, Su T-P, Tsai S-J, Li C-T, Lin W-C, Chang W-H, Pan T-L et al (2016) Influence of relative age on diagnosis and treatment of attention-deficit hyperactivity disorder in Taiwanese children. J Pediatr 172:162–167 26973148 10.1016/j.jpeds.2016.02.012

[CR23] Chorniy A, Kitashima L (2016) Sex, drugs, and ADHD: the effects of ADHD pharmacological treatment on teens’ risky behaviors. Labour Econ 43:87–105

[CR24] Cornelissen T, Dustmann C (2019) Early school exposure, test scores, and noncognitive outcomes. Am Econ J Econ Pol 11(2):35–63

[CR25] Coupland C, Harcourt S, Vinogradova Y, Smith G, Joseph C, Pringle M, Hippisley-Cox J (2007) Inequalities in uptake of influenza vaccine by deprivation and risk group: time trends analysis. Vaccine 25(42):7363–7371 17884258 10.1016/j.vaccine.2007.08.032

[CR26] Crawford C, Dearden L, Greaves E (2013) The impact of age within academic year on adult outcomes. Technical report, IFS Working Papers

[CR27] Crawford C, Dearden L, Greaves E (2014) The drivers of month-of-birth differences in children’s cognitive and non-cognitive skills. J R Stat Soc A Stat Soc 177(4):829–860 10.1111/rssa.12071 PMC4282424 25598586

[CR28] Cuddy E, Currie J (2020) Rules vs. discretion: Treatment of mental illness in us adolescents. Technical report, National Bureau of Economic Research, forthcoming Journal of Political Economy

[CR29] Currie J, Stabile M (2006) Child mental health and human capital accumulation: the case of ADHD. J Health Econ 25(6):1094–1118 16730082 10.1016/j.jhealeco.2006.03.001

[CR30] Currie J, Zwiers E (2025) Medication of postpartum depression and maternal outcomes. Journal of Human Resources 60(4):1093–1125

[CR31] Currie J, Stabile M, Manivong P, Roos LL (2010) Child health and young adult outcomes. Journal of Human Resources 45(3):517–548

[CR32] Dalsgaard S, Humlum MK, Nielsen HS, Simonsen M (2012) Relative standards in ADHD diagnoses: The role of specialist behavior. Econ Lett 117(3):663–665

[CR33] Currie J, Stabile M, Jones L (2014) Do stimulant medications improve educational and behavioral outcomes for children with ADHD? J Health Econ 37:58–69 10.1016/j.jhealeco.2014.05.002 PMC4815037 24954077

[CR34] Dalsgaard S, Humlum MK, Nielsen HS, Simonsen M (2014a) Common Danish standards in prescribing medication for children and adolescents with ADHD. European Child & Adolescent Psychiatry 23:841–844 10.1007/s00787-013-0508-5 24374648

[CR35] Dalsgaard S, Nielsen HS, Simonsen M (2014b) Consequences of ADHD medication use for children’s outcomes. J Health Econ 37:137–151 10.1016/j.jhealeco.2014.05.005 24997381

[CR36] Dettling AC, Gunnar MR, Donzella B (1999) Cortisol levels of young children in full-day childcare centers: Relations with age and temperament. Psychoneuroendocrinology 24(5):519–536 10378239 10.1016/s0306-4530(99)00009-8

[CR37] Dhuey E, Figlio D, Karbownik K, Roth J (2019) School starting age and cognitive development. J Policy Anal Manage 38(3):538–578

[CR38] Ding W, Lehrer SF, Rosenquist JN, Audrain-McGovern J (2009) The impact of poor health on academic performance: New evidence using genetic markers. J Health Econ 28(3):578–597 19217678 10.1016/j.jhealeco.2008.11.006

[CR39] Efron D (2017) The role of schools in the diagnosis of adhd. The Lancet Psychiatry 4(11):825–826 29033004 10.1016/S2215-0366(17)30406-6

[CR40] Einav L, Finkelstein A, Oostrom T, Ostriker A, Williams H (2020) Screening and selection: The case of mammograms. American Economic Review 110(12):3836–3870 34305149 10.1257/aer.20191191 PMC8300583

[CR41] Elder TE (2010) The importance of relative standards in ADHD diagnoses: Evidence based on exact birth dates. J Health Econ 29(5):641–656 10.1016/j.jhealeco.2010.06.003 PMC2933294 20638739

[CR42] Elder TE, Lubotsky DH (2009) Kindergarten entrance age and children’s achievement: Impacts of state policies, family background, and peers. Journal of Human Resources 44(3):641–683

[CR43] Elder T, Zhou Y (2021) The black-white gap in noncognitive skills among elementary school children. Am Econ J Appl Econ 13(1):105–32

[CR44] Emma Degroote M-C B, Houtte MV (2022) Suspicion of ADHD by teachers in relation to their perception of students’ cognitive capacities: do cognitively strong students escape verdict?. Int J Inclusive Educ 0(0):1–15

[CR45] Evans WN, Morrill MS, Parente ST (2010) Measuring inappropriate medical diagnosis and treatment in survey data: The case of ADHD among school-age children. J Health Econ 29(5):657–673 20739076 10.1016/j.jhealeco.2010.07.005

[CR46] Faraone SV, Sergeant J, Gillberg C, Biederman J (2003) The worldwide prevalence of ADHD: Is it an American condition? World Psychiatry 2(2):104 PMC1525089 16946911

[CR47] Fleming M, Bandyopadhyay A, McLay JS, Clark D, King A, Mackay DF, Lyons RA, Sayal K, Brophy S, Pell JP (2022) Age within schoolyear and attention-deficit hyperactivity disorder in Scotland and Wales. BMC Public Health 22(1):1–9 35637502 10.1186/s12889-022-13453-w PMC9150337

[CR48] Fletcher JM (2014) The effects of childhood ADHD on adult labor market outcomes. Health Econ 23(2):159–181 10.1002/hec.2907 PMC6714576 23427026

[CR49] Fletcher J, Wolfe B (2008) Child mental health and human capital accumulation: The case of ADHD revisited. J Health Econ 27(3):794–800 18221807 10.1016/j.jhealeco.2007.10.010

[CR50] Fletcher J, Wolfe B (2009) Long-term consequences of childhood ADHD on criminal activities. J Ment Health Policy Econ 12(3):119 19996475 PMC3398051

[CR51] Frisira E, Holland J, Sayal K (2025) Systematic review and meta-analysis: relative age in attention-deficit/hyperactivity disorder and autism spectrum disorder. European Child & Adolescent Psychiatry 34:381–401 38767699 10.1007/s00787-024-02459-x PMC11868292

[CR52] Furzer J, Dhuey E, Laporte A (2022) ADHD misdiagnosis: Causes and mitigators. Health Econ 31(9):1926–1953 35763436 10.1002/hec.4555

[CR53] Gao M, Piernas C, Astbury NM, Hippisley-Cox J, O’Rahilly S, Aveyard P, Jebb SA (2021) Associations between body-mass index and COVID-19 severity in 6.9 million people in England: a prospective, community-based, cohort study. The Lancet Diabetes & Endocrinology 9(6):350–359 10.1016/S2213-8587(21)00089-9 PMC8081400 33932335

[CR54] Gould MS, Walsh BT, Munfakh JL, Kleinman M, Duan N, Olfson M, Greenhill L, Cooper T (2009) Sudden death and use of stimulant medications in youths. Am J Psychiatry 166(9):992–1001 19528194 10.1176/appi.ajp.2009.09040472

[CR55] Gunnar M, Quevedo K (2007) The neurobiology of stress and development. Annu Rev Psychol 58(1):145–173 16903808 10.1146/annurev.psych.58.110405.085605

[CR56] Hartman CA, Rommelse N, van der Klugt CL, Wanders RB, Timmerman ME (2019) Stress exposure and the course of ADHD from childhood to young adulthood: Comorbid severe emotion dysregulation or mood and anxiety problems. J Clin Med 8(11):1824 31683870 10.3390/jcm8111824 PMC6912831

[CR57] Hausman C, Rapson DS (2018) Regression discontinuity in time: Considerations for empirical applications. Ann Rev Res Econ 10(1):533–552

[CR58] Hippisley-Cox J, Coupland C (2010) Unintended effects of statins in men and women in England and Wales: population based cohort study using the QResearch database. BMJ 340:c2197 20488911 10.1136/bmj.c2197 PMC2874131

[CR59] Hippisley-Cox J, Coupland C, Brindle P (2014) The performance of seven QPrediction risk scores in an independent external sample of patients from general practice: a validation study. BMJ Open 4(8):e005809 10.1136/bmjopen-2014-005809 PMC4156807 25168040

[CR60] Hippisley-Cox J, Vinogradova Y, Coupland C, Pringle M (2005) Comparison of key practice characteristics between general practices in England and Wales and general practices in the QRESEARCH data

[CR61] Humphreys KL, Watts EL, Dennis EL, King LS, Thompson PM, Gotlib IH (2019) Stressful life events, ADHD symptoms, and brain structure in early adolescence. J Abnorm Child Psychol 47:421–432 29785533 10.1007/s10802-018-0443-5 PMC6249129

[CR62] Jepsen JRM, Michel M (2006) ADHD and the symptom dimensions inattention, impulsivity, and hyperactivity: A review of aetiological twin studies from 1996 to 2004. Nordic Psychology 58(2):108–135

[CR63] Kiessling L, Norris J (2023) The long-run effects of peers on mental health. Econ J 133(649):281–322

[CR64] Krabbe E, Thoutenhoofd E, Conradi M, Pijl S, Batstra L (2014) Birth month as predictor of ADHD medication use in dutch school classes. Eur J Spec Needs Educ 29(4):571–578

[CR65] Layton TJ, Barnett ML, Hicks TR, Jena AB (2018) Attention deficit-hyperactivity disorder and month of school enrollment. N Engl J Med 379(22):2122–2130 30485780 10.1056/NEJMoa1806828 PMC6322209

[CR66] Livingstone LT, Coventry WL, Corley RP, Willcutt EG, Samuelsson S, Olson RK, Byrne B (2016) Does the environment have an enduring effect on ADHD? A longitudinal study of monozygotic twin differences in children. J Abnorm Child Psychol 44(8):1487–1501 26993487 10.1007/s10802-016-0145-9 PMC5027180

[CR67] Mannuzza S, Klein RG (2000) Long-term prognosis in attention-deficit/hyperactivity disorder. Child Adolesc Psychiatr Clin N Am 9(3):711–726 10944664

[CR68] Marquardt K (2025) Mis(sed) diagnosis: Physician decision-making and ADHD. FRB of Chicago Working Paper. Federal Reserve of Chicago

[CR69] McEwan PJ, Shapiro JS (2008) The benefits of delayed primary school enrollment: Discontinuity estimates using exact birth dates. Journal of Human Resources 43(1):1–29

[CR70] McKechnie DG, O’Nions E, Dunsmuir S, Petersen I (2023) Attention-deficit hyperactivity disorder diagnoses and prescriptions in UK primary care, 2000–2018: population-based cohort study. BJPsych Open 9(4):e121 37455585 10.1192/bjo.2023.512 PMC10375867

[CR71] Morrow RL, Garland EJ, Wright JM, Maclure M, Taylor S, Dormuth CR (2012) Influence of relative age on diagnosis and treatment of attention-deficit/hyperactivity disorder in children. Can Med Assoc J 184(7):755–762 22392937 10.1503/cmaj.111619 PMC3328520

[CR72] Nafilyan V, Humberstone B, Mehta N, Diamond I, Coupland C, Lorenzi L, Pawelek P, Schofield R, Morgan J, Brown P et al (2021) An external validation of the QCovid risk prediction algorithm for risk of mortality from COVID-19 in adults: A national validation cohort study in England. The Lancet Digital Health 3(7):e425–e433 34049834 10.1016/S2589-7500(21)00080-7 PMC8148652

[CR73] NICE (2018) Attention deficit hyperactivity disorder: Diagnosis and management 29634174

[CR74] Nicoletti C, Vidiella-Martin J (2025) ADHD, school performance, and economic outcomes. In Oxford Research Encyclopedia of Economics and Finance. Oxford University Press

[CR75] Öster C, Ramklint M, Meyer J, Isaksson J (2020) How do adolescents with ADHD perceive and experience stress? An interview study. Nord J Psychiatry 74(2):123–130 31613179 10.1080/08039488.2019.1677771

[CR76] Patel DR, Feucht C, Brown K, Ramsay J (2018) Pharmacological treatment of anxiety disorders in children and adolescents: A review for practitioners. Translational Pediatrics 7(1):23 29441280 10.21037/tp.2017.08.05 PMC5803020

[CR77] Persson P, Qiu X, Rossin-Slater M (2025) Family spillover effects of marginal diagnoses: The case of ADHD. Am Econ J Appl Econ 17(2):225–256

[CR78] Pottegård A, Hallas J, Zoëga H (2014) Children’s relative age in class and use of medication for ADHD: a Danish Nationwide Study. J Child Psychol Psychiatry 55(11):1244–1250 24813478 10.1111/jcpp.12243 PMC4277337

[CR79] Riglin L, Collishaw S, Thapar AK, Dalsgaard S, Langley K, Smith GD, Stergiakouli E, Maughan B, O’Donovan MC, Thapar A (2016) Association of genetic risk variants with attention-deficit/hyperactivity disorder trajectories in the general population. JAMA Psychiat 73(12):1285–1292 10.1001/jamapsychiatry.2016.2817 PMC6485350 27806167

[CR80] Rimm-Kaufman SE, Pianta RC (2000) An ecological perspective on the transition to kindergarten: A theoretical framework to guide empirical research. J Appl Dev Psychol 21(5):491–511

[CR81] Robertson E (2011) The effects of quarter of birth on academic outcomes at the elementary school level. Econ Educ Rev 30(2):300–311

[CR82] Rockhill C, Kodish I, DiBattisto C, Macias M, Varley C, Ryan S (2010) Anxiety disorders in children and adolescents. Curr Probl Pediatr Adolesc Health Care 40(4):66–99 20381781 10.1016/j.cppeds.2010.02.002

[CR83] Root A, Brown JP, Forbes HJ, Bhaskaran K, Hayes J, Smeeth L, Douglas IJ (2019) Association of relative age in the school year with diagnosis of intellectual disability, attention-deficit/hyperactivity disorder, and depression. JAMA Pediatr 173(11):1068–1075 31545342 10.1001/jamapediatrics.2019.3194 PMC6763997

[CR84] Sarzosa M, Urzúa S (2021) Bullying among adolescents: The role of skills. Quant Econ 12(3):945–980

[CR85] Scahill L, Schwab-Stone M (2000) Epidemiology of ADHD in school-age children. Child Adolesc Psychiatr Clin N Am 9(3):541–555 10944656

[CR86] Schnorrbusch C, Fabiano GA, Aloe AM, Toro Rodriguez RC (2020) Attention deficit hyperactivity disorder and relative age: A meta-analysis. Sch Psychol Rev 49(1):2–19

[CR87] Schwandt H, Wuppermann A (2016) The youngest get the pill: ADHD misdiagnosis in Germany, its regional correlates and international comparison. Labour Econ 43:72–86

[CR88] Setlik J, Bond GR, Ho M (2009) Adolescent prescription ADHD medication abuse is rising along with prescriptions for these medications. Pediatrics 124(3):875–880 19706567 10.1542/peds.2008-0931

[CR89] Shigeoka H (2015) School entry cutoff date and the timing of births. NBER Working Paper Series 21402, National Bureau of Economic Research

[CR90] Singh I (2008) ADHD, culture and education. Early Child Dev Care 178(4):347–361

[CR91] Thomas R, Sanders S, Doust J, Beller E, Glasziou P (2015) Prevalence of attention-deficit/hyperactivity disorder: A systematic review and meta-analysis. Pediatrics 135(4):e994–e1001 25733754 10.1542/peds.2014-3482

[CR92] Toole KP, Frank C (2024) A young adolescent with undiagnosed ADHD-inattentive presentation and co-morbid anxiety and depression: A case report. J Pediatr Nurs 78:e250–e259 39127589 10.1016/j.pedn.2024.07.013

[CR93] Townsend P, Phillimore P, Beattie A (1988) Health and deprivation: Inequality and the North. Routledge

[CR94] Whitely M, Raven M, Timimi S, Jureidini J, Phillimore J, Leo J, Moncrieff J, Landman P (2018) Attention deficit hyperactivity disorder late birthdate effect common in both high and low prescribing international jurisdictions: Systematic review. J Child Psychol Psychiatry 60(4):380–391 30317644 10.1111/jcpp.12991 PMC7379308

[CR95] Zoëga H, Valdimarsdóttir UA, Hernández-Díaz S (2012) Age, academic performance, and stimulant prescribing for ADHD: A nationwide cohort study. Pediatrics 130(6):1012–1018 23166340 10.1542/peds.2012-0689 PMC3507253

