# Supplementary material for: Why are the youngest in class more often prescribed pharmacological treatment for ADHD?
Source: J Popul Econ. 2026 Jul 23;39(3):45. doi: 10.1007/s00148-026-01190-y (PMC13391727; doi:10.1007/s00148-026-01190-y)
Supplement: Supplementary file 1 — (pdf 955 KB) [file 148_2026_1190_MOESM1_ESM.pdf]

Supplementary Online Appendix to

"Why are the youngest in class more often prescribed pharmacological treatment for ADHD? "

(Catia Nicodemo, Cheti Nicoletti, and Joaquim Vidiella-Martin)

For Online Publication Only

Appendix A: Additional figures and tables

Figure A.1: Rate of ADHD prescriptions by age and school starting age, including middle starters

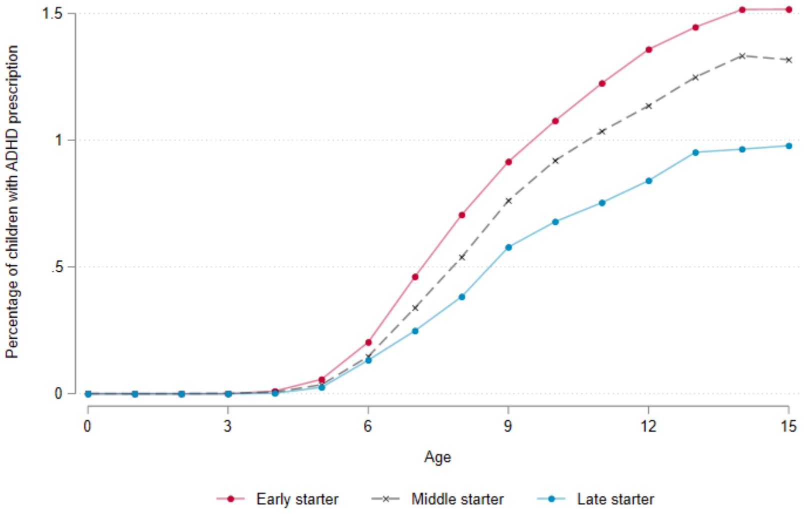

Notes: Each marker captures the percentage of children receiving an ADHD prescription in the corresponding age period. The figure complements Figure 1b, which considers early starters (born in July and August) and late starters (born in September and October) separately and includes middle starters (born between November and June. As reported in Table 2, our main sample of early and late starters consists of $N = 96,698$ individuals (49,157 early starters and 47,541 late starters). Additionally, the figure above includes $N = 196,938$ middle starters, who are not part of our main analyses.

Figure A.2: Manipulation testing: estimated density of the running variable

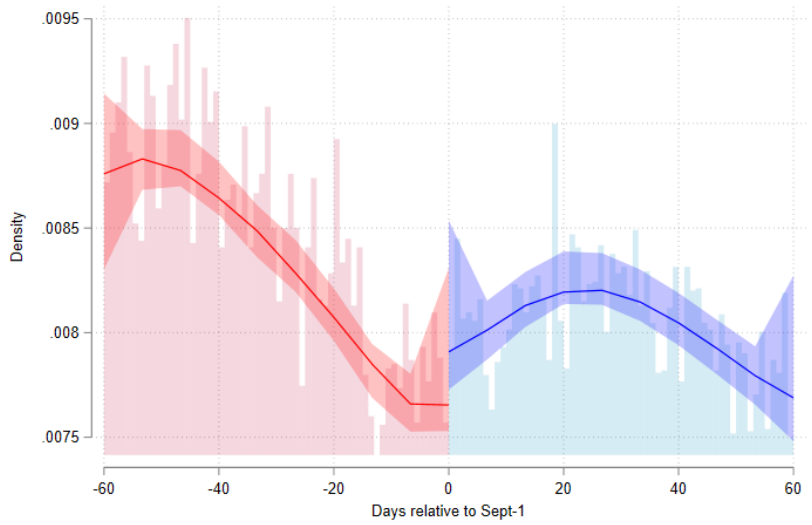

Notes: The horizontal axis depicts the date of birth relative to the cut-off point at school entry of September 1. The solid lines depict the local polynomial density estimate (blue and red on each side of the September 1 cutoff). The shaded areas capture the robust bias-corrected confidence intervals. The histogram of the running variable, the children's birth date in days centered around the cutoff for school entry, is shown in the background.

Figure A.3: Rate of ADHD prescriptions for late starters by age and grade

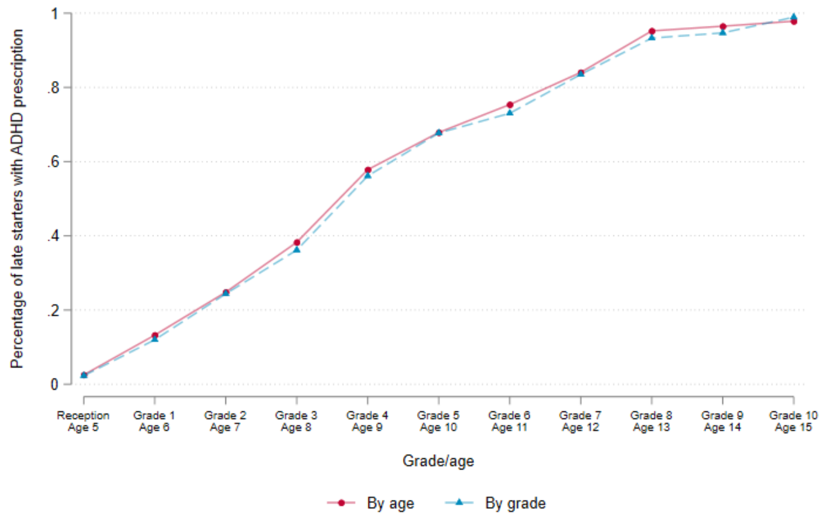

Notes: Each marker captures the percentage of late starters (i.e., born in September and October) receiving an ADHD prescription in the corresponding age (from ages 5 to 15) and grade period (from reception class to grade 10).

Figure A.4: Rate of continuation of ADHD prescriptions by age, conditional on receiving at least one prescription before age 9

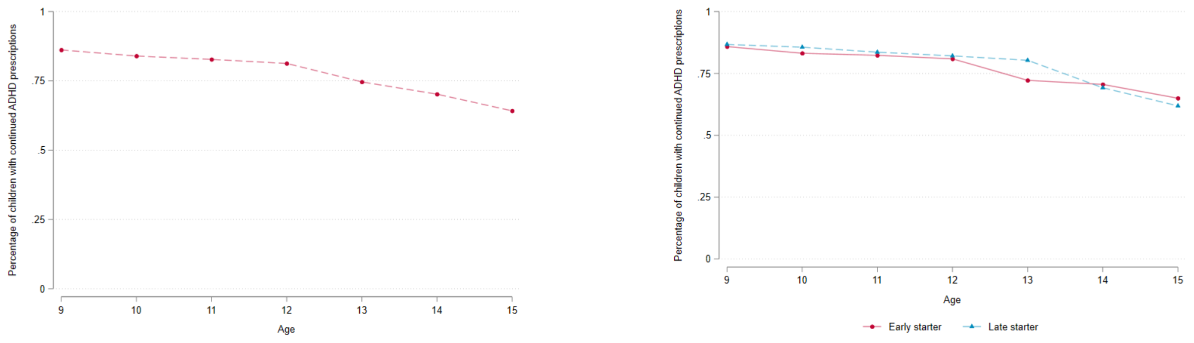

(a) Pooled sample

(b) Early vs late starters

Notes: Each marker captures the percentage of children receiving an ADHD prescription in the corresponding age period, conditional on having received at least one prescription between ages 5 and 8. Panel (a) considers the full sample of children born between July and October, while Panel (b) considers early starters (born in July and August) and late starters (born in September and October) separately.

Figure A.5: Histogram of the age at which children receive their first ADHD prescription

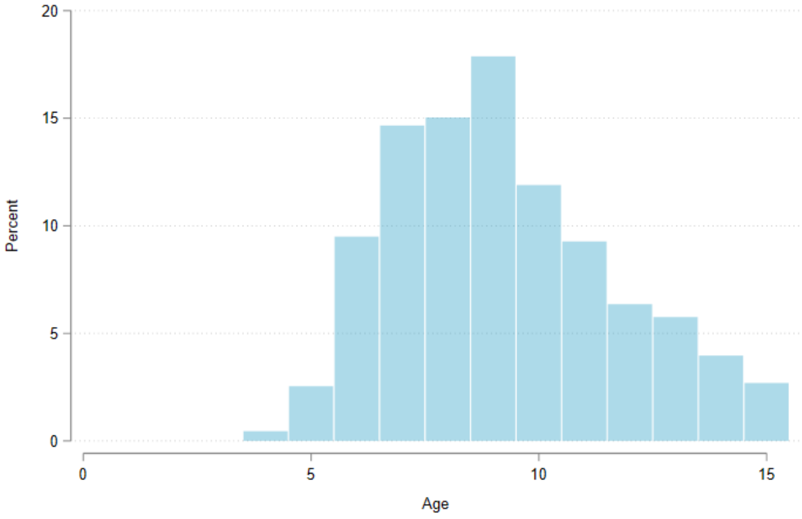

Notes: To construct this figure, we consider a sub-sample of children who received at least one ADHD prescription before age 15 and plot the distribution of the age at which these children received their first prescription ($n = 1,337$). 6 of these children received their first prescription at age 4, against the recommended guidelines.

Figure A.6: Effect of early start of school on ADHD prescriptions by age, including maternal fixed effects

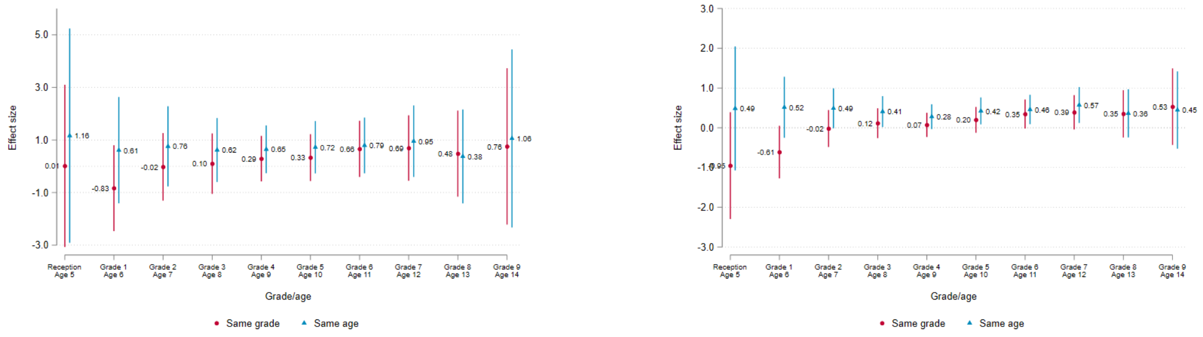

(a) Bandwidth = 60 days

(b) Bandwidth = 120 days

Notes: The figure plots point estimates and 95% confidence intervals of separate regressions, one for each age from 5 to 15 and grade from reception class to grade 10. The effects are expressed as a proportional increase in the prescription rate for early starters relative to late starters, $r_t = \gamma_t / Pr(ADHD_{i,t} = 1 | Early_i = 0)$ with $t = a$ for age and g for grade. In each regression, we control for sex and include mother and year of birth fixed effects. Panel (a) uses all sibling combinations in a sample of children born within 60 days of the September 1 cutoff date. In Panel (b), we expand this bandwidth to 120 days at either side of the September 1 cutoff point. The effective sample sizes are reported in Table A.4.

Figure A.7: Effect of early start of school on ADHD prescriptions by age, using alternative measurements of date of birth

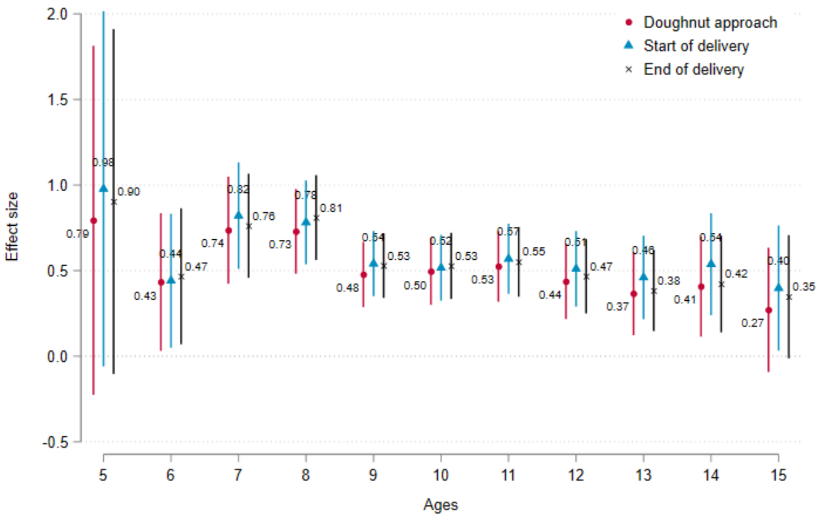

Notes: The figure plots point estimates and 95% confidence intervals of separate regressions, one for each age from 5 to 15. The effects are expressed as a proportional increase in the prescription rate for early starters relative to late starters, $r_a = \gamma_a / Pr(ADHD_{i,a} = 1 | Early_i = 0)$. In each regression, we control for sex, ethnicity, postal code SES, and maternal age at birth, and include general practice and year of birth fixed effects. Compared to the results in Figure 4, we show three different models: (1) excluding children born within ± 3 days of the September 1 threshold, (2) using the start of the maternity inpatient care admission spell as the birthdate, and (3) using the end of such spell as the birthdate.

Figure A.8: Effect of early start of school on ADHD prescriptions by age, using a logistic model

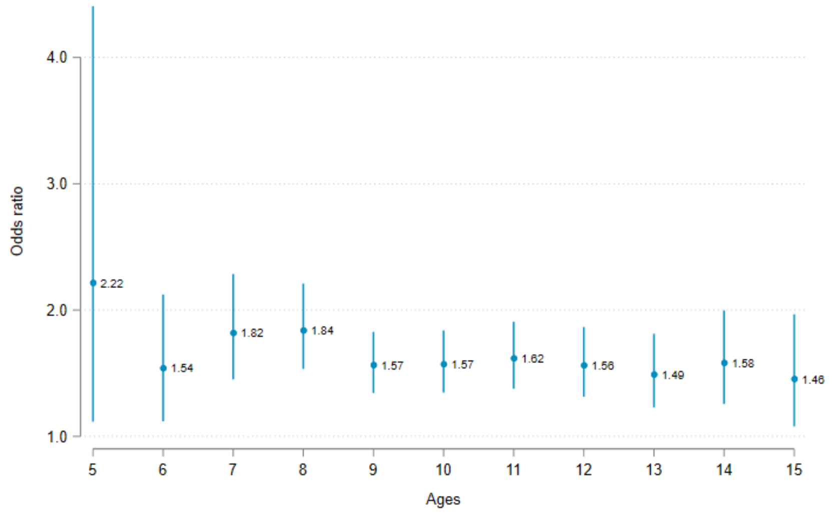

Notes: The figure plots point estimates and 95% confidence intervals of separate regressions, one for each age from 5 to 15. The effects are expressed as odds ratios. In each regression, we control for sex, ethnicity, postal code SES, and maternal age at birth, and include general practice and year of birth fixed effects.

Figure A.9: Rate of ADHD prescriptions by cohort

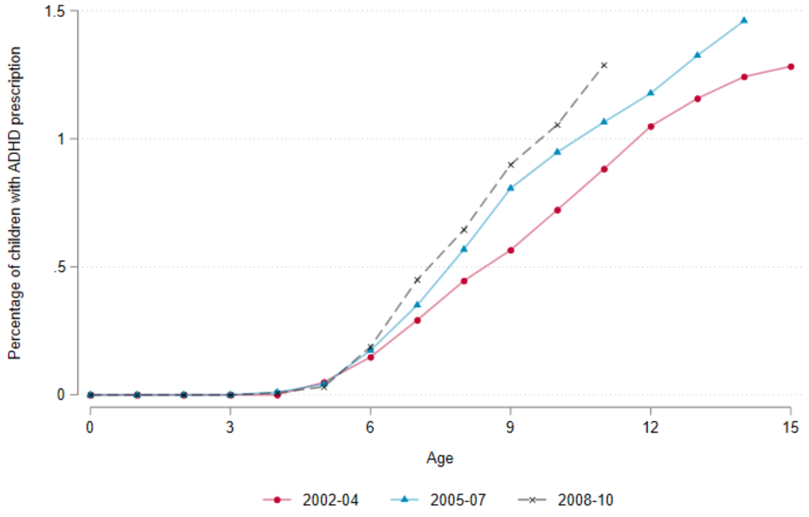

Notes: Each marker captures the percentage of children receiving an ADHD prescription in the corresponding age period. Since we only observe children until 2020, children born in later cohorts (in blue and black) cannot be tracked until age 15.

Figure A.10: Effect of early start of school on ADHD prescriptions by age and cohort

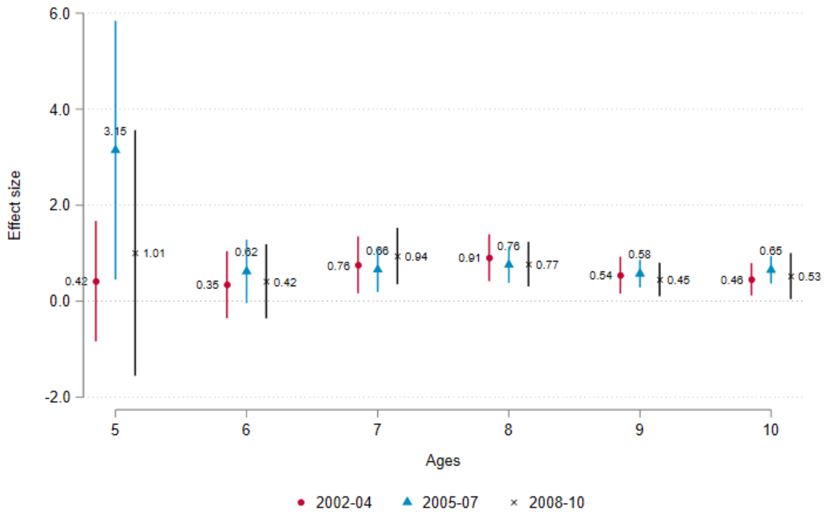

Notes: The figure plots point estimates and 95% confidence intervals of separate regressions, one for each age from 5 to 10, and separately for three different birth cohorts. The effects are expressed as a proportional increase in the prescription rate for early starters relative to late starters, $r_a = \gamma_a / Pr(ADHD_{i,a} = 1|Early_i = 0)$. In each regression, we control for sex, ethnicity, postal code SES, and maternal age at birth, and include general practice and year of birth fixed effects.

Table A.1: Decomposition of the early-starter effect and identification of mechanisms

Object	Eq.	Expression	Interpretation
Early-starter effect	(2)	$\gamma_t = \gamma_{EXP,t} - \gamma_{AGEE,t} - \gamma_{RELAGE,t} - \gamma_{ABSAGE,t}$	Total effect of being an early starter, combining all mechanisms.
Same-grade effect	(3)	$\gamma_g = -\gamma_{AGEE,g} - \gamma_{RELAGE,g} - \gamma_{ABSAGE,g}$	Effect of early entry holding grade constant; excludes length of school exposure.
Same-age effect	(4)	$\gamma_a = \gamma_{EXP,a} - \gamma_{AGEE,a} - \gamma_{RELAGE,a}$	Effect of early entry holding age constant; excludes absolute age.
Difference	(5)	$\gamma_a - \gamma_g = \gamma_{EXP,a} + \gamma_{ABSAGE,g}$	Isolates the combined contribution of length of school exposure and absolute age.

Notes: The table summarizes how the effect of being an early starter is decomposed into underlying mechanisms and how these are identified using same-age and same-grade comparisons.

Table A.2: Testing differences between same age and same grade estimates on the effect of early start of school on anxiety prescriptions

Age/grade	Same age (1)	Same grade (2)	p-value (3)
Age 5/Reception	0.018	-0.046	0.077
	(0.025)	(0.022)	
	[0.141]	[-0.368]	
Age 6/Grade 1	0.089	-0.024	0.055
	(0.031)	(0.027)	
	[0.510]	[-0.140]	
Age 7/Grade 2	0.076	0.005	0.114
	(0.037)	(0.034)	
	[0.271]	[0.018]	
Age 8/Grade 3	0.102	-0.008	0.087
	(0.043)	(0.040)	
	[0.267]	[-0.022]	
Age 9/Grade 4	0.062	-0.010	0.155
	(0.048)	(0.046)	
	[0.123]	[-0.020]	
Age 10/Grade 5	0.108	-0.033	0.094
	(0.058)	(0.054)	
	[0.184]	[-0.057]	
Age 11/Grade 6	0.125	0.004	0.131
	(0.069)	(0.065)	
	[0.184]	[0.006]	
Age 12/Grade 7	-0.006	-0.081	0.242
	(0.082)	(0.079)	
	[-0.007]	[-0.102]	
Age 13/Grade 8	-0.027	-0.107	0.268
	(0.099)	(0.094)	
	[-0.031]	[-0.127]	
Age 14/Grade 9	-0.081	-0.082	0.985
	(0.123)	(0.120)	
	[-0.087]	[-0.090]	
Age 15/Grade 10	0.011	-0.124	0.236
	(0.162)	(0.154)	
	[0.011]	[-0.137]	

Notes: The table reports the point estimates, standard errors (in parentheses), and effect sizes (in brackets) of separate regressions, one for each age from 5 to 15 (column 1) and grade from reception class to grade 10 (column 2). The estimates and standard errors are expressed in absolute values, while the effect sizes are expressed as a proportional increase in the prescription rate for early starters relative to late starters, $r_t = \gamma_t / Pr(Anxiety_{i,t} = 1 | Early_i = 0)$ with $t = a$ for age and g for grade. These estimates are the same as the ones reported in Figure 7a. In each regression, we control for sex, ethnicity, postal code SES, and maternal age at birth, and include general practice and year of birth fixed effects. In column 3, we report the p-values for a test of the difference between the results in columns 1 and 2, assuming the null hypothesis of no difference between the estimates.

Table A.3: Testing differences between same age and same grade estimates on the effect of early start of school on anxiety diagnoses

Age/grade	Same age (1)	Same grade (2)	p-value (3)
Age 5/Reception	0.036	-0.056	0.061
	(0.031)	(0.027)	
	[0.184]	[-0.289]	
Age 6/Grade 1	0.076	-0.050	0.057
	(0.039)	(0.034)	
	[0.252]	[-0.174]	
Age 7/Grade 2	0.098	-0.061	0.060
	(0.047)	(0.043)	
	[0.214]	[-0.137]	
Age 8/Grade 3	0.085	-0.068	0.073
	(0.056)	(0.052)	
	[0.124]	[-0.104]	
Age 9/Grade 4	0.048	-0.116	0.079
	(0.064)	(0.060)	
	[0.054]	[-0.133]	
Age 10/Grade 5	0.173	-0.131	0.052
	(0.078)	(0.071)	
	[0.161]	[-0.126]	
Age 11/Grade 6	0.341	-0.017	0.053
	(0.096)	(0.088)	
	[0.269]	[-0.013]	
Age 12/Grade 7	-0.067	-0.230	0.151
	(0.119)	(0.114)	
	[-0.039]	[-0.137]	
Age 13/Grade 8	-0.158	-0.605	0.069
	(0.157)	(0.144)	
	[-0.070]	[-0.281]	
Age 14/Grade 9	0.025	-0.698	0.056
	(0.217)	(0.199)	
	[0.009]	[-0.254]	
Age 15/Grade 10	0.200	-0.689	0.062
	(0.315)	(0.288)	
	[0.058]	[-0.204]	

Notes: The table reports the point estimates, standard errors (in parentheses), and effect sizes (in brackets) of separate regressions, one for each age from 5 to 15 (column 1) and grade from reception class to grade 10 (column 2). The estimates and standard errors are expressed in absolute values, while the effect sizes are expressed as a proportional increase in the diagnosis rate for early starters relative to late starters, $r_t = \gamma_t / Pr(Anxiety_{i,t} = 1 | Early_i = 0)$ with $t = a$ for age and g for grade. These estimates are the same as the ones reported in Figure 7b. In each regression, we control for sex, ethnicity, postal code SES, and maternal age at birth, and include general practice and year of birth fixed effects. In column 3, we report the p-values for a test of the difference between the results in columns 1 and 2, assuming the null hypothesis of no difference between the estimates.

Table A.4: Number of observations by age when including mother fixed effects

Age	Observations with bandwidth = 60	Observations with bandwidth = 120
5	13,578	49,322
6	13,578	49,322
7	13,578	49,322
8	13,578	49,322
9	13,578	49,322
10	10,448	38,599
11	7,634	28,937
12	5,155	19,932
13	3,040	12,201
14	1,428	6,076
15	405	1,887

Table A.5: Effect of early start of school on ever being hospitalized for placebo outcomes

	Gastroenteritis	Chest pain	Epilepsy	Ear infections	Asthma	Flu	Pneumonia	Conjunctivitis	Any admission
	(1)	(2)	(3)	(4)	(5)	(6)	(7)	(8)	(9)
Early starter	0.001	0.000	-0.001	0.001	-0.003**	0.000	-0.001	-0.000	0.005
	(0.001)	(0.000)	(0.000)	(0.001)	(0.001)	(0.000)	(0.000)	(0.000)	(0.003)
N	96,698	96,698	96,698	96,698	96,698	96,698	96,698	96,698	96,698
Mean DV for late starters	0.006	0.004	0.005	0.017	0.039	0.001	0.005	0.001	0.328
Effect size	0.130	0.082	-0.136	0.073	-0.064	0.025	-0.122	-0.171	0.015

Notes: The figure reports the estimated γ_{15} in equation (6) when the dependent variable is an indicator taking value 1 if the individual experienced at least one hospital admission from age 5 up to age 15 for the specified placebo outcome. The effect size is expressed as the proportional increase in the rate of ever being hospitalized for early starters relative to late starters, $r_{15} = \gamma_{15}/Pr(EverHosp_{i,15} = 1|Early_i = 0)$. Placebo outcomes include malnutrition, gastroenteritis, chest pain, epilepsy, ear infections, asthma, flu, pneumonia, conjunctivitis, and any hospital admission, constructed using Hospital Episode Statistics (HES) data. We control for sex, ethnicity, postal code SES, and maternal age at birth, and include general practice and year of birth fixed effects.

Table A.6: Effect of early start of school on ever being prescribed ADHD pharmacological treatment, weighted by exposure

	Ever prescribed ADHD drugs
	(1)
Early start	0.007***
	(0.001)
N	96,698
Mean ever being prescribed for late starters, $Pr(EverADHD_{i,15} = 1\|Early_i = 0)$	0.011
Effect size, $\gamma_{15}/Pr(EverADHD_{i,15} = 1\|Early_i = 0)$	0.600
Weighting	By exposure

Notes: The table reports the estimated γ_{15} in equation (6) when the dependent variable is an indicator taking value 1 if the individual received at least one ADHD drug prescription up to age 15. The effect size is expressed as the proportional increase in the rate of having ever been treated with pharmacological treatment for early starters relative to late starters, $r_{15} = \gamma_g/Pr(EverADHD_{i,15} = 1|Early_i = 0)$. We control for sex, ethnicity, postal code SES, and maternal age at birth, and include general practice and year-of-birth fixed effects. Individuals are weighted proportionally to the total number of years they are observed.

Table A.7: Average marginal effects of early school entry with GP practice interactions

	Ever prescribed ADHD drugs			
	(1)	(2)	(3)	(4)
Early starter	0.006***	0.006*	0.005***	0.007***
	(0.001)	(0.003)	(0.001)	(0.001)
Interaction	Baseline model	Early x GP	Early x GP	Early x GP
Practices with at least N children		200	100	50
N children	96,698	7,285	40,251	76,102
N practices	1,740	28	273	778
P-val of H0: interactions are jointly zero		0.307	0.734	1.000
Mean DV for late starters, $Pr(EverADHD_{i,15} = 1 \mid Early_i = 0)$	0.011	0.013	0.012	0.011
Effect size, $\gamma_{15}/Pr(EverADHD_{i,15} = 1 \mid Early_i = 0)$	0.611	0.491	0.405	0.670

Notes: Column (1) reports the marginal effect of early school entry from the baseline specification without interactions, estimated using all general practices. Columns (2)-(4) report average marginal effects from specifications that interact the early-entry indicator with general practice fixed effects, restricting the sample to practices with at least 200, 100, and 50 observations, respectively. In the presence of these interactions, the coefficient on the early-entry indicator is no longer directly interpretable, so effects are summarized using average marginal effects. All specifications include the same controls as in the baseline analysis, namely sex, ethnicity, postal code socioeconomic status, maternal age at birth, and year-of-birth fixed effects.

Appendix B: Using ADHD diagnoses instead of pharmacological treatment

Figure B.1: Average rate of ADHD diagnoses by age

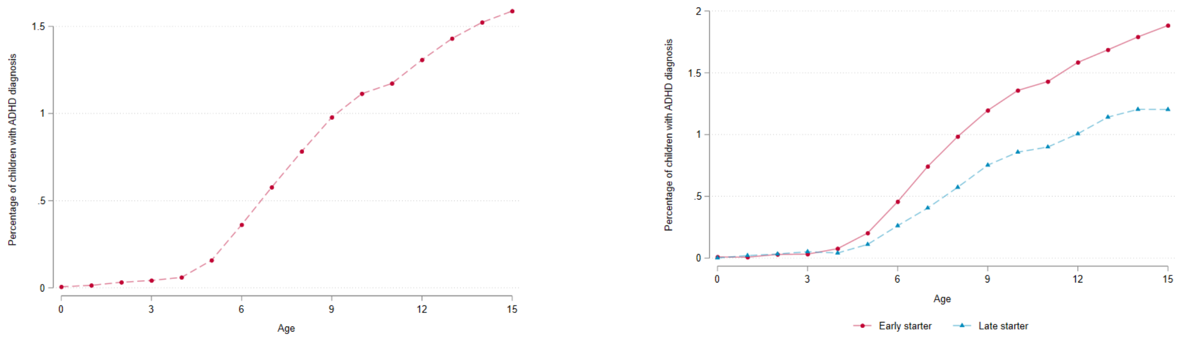

(a) Pooled sample **(b)** By school starting age

Notes: Each marker captures the percentage of children diagnosed with ADHD in the corresponding age period.

Figure B.2: Effect of early start of school on ADHD diagnoses by age and grade

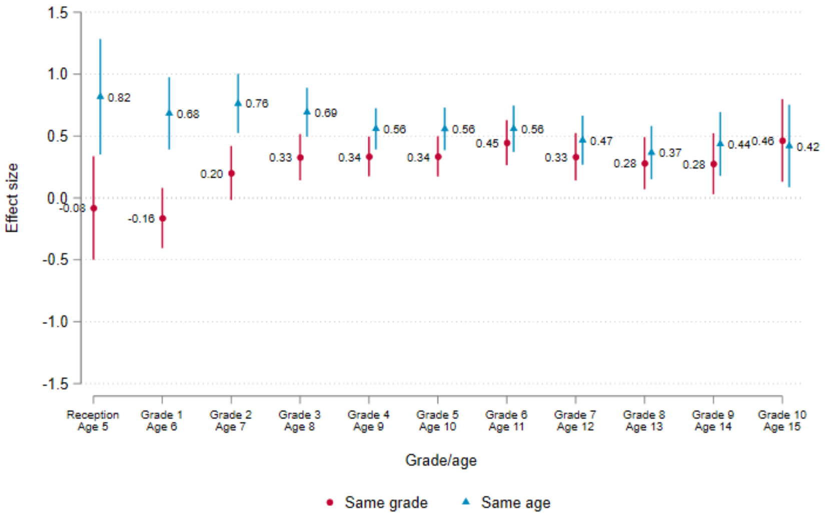

Notes: The figure plots point estimates and 95% confidence intervals of separate regressions, one for each age from 5 to 15 and grade from reception class to grade 10. The effects are expressed as a proportional increase in the diagnosis rate for early starters relative to late starters, $r_t = \gamma_t / Pr(ADHD_{i,t} = 1 | Early_i = 0)$ with $t = a$ for age and g for grade. We control for sex, ethnicity, postal code SES, and maternal age at birth, and include general practice and year of birth fixed effects.

Figure B.3: Effect of early start of school on ADHD first-time diagnoses by age and grade

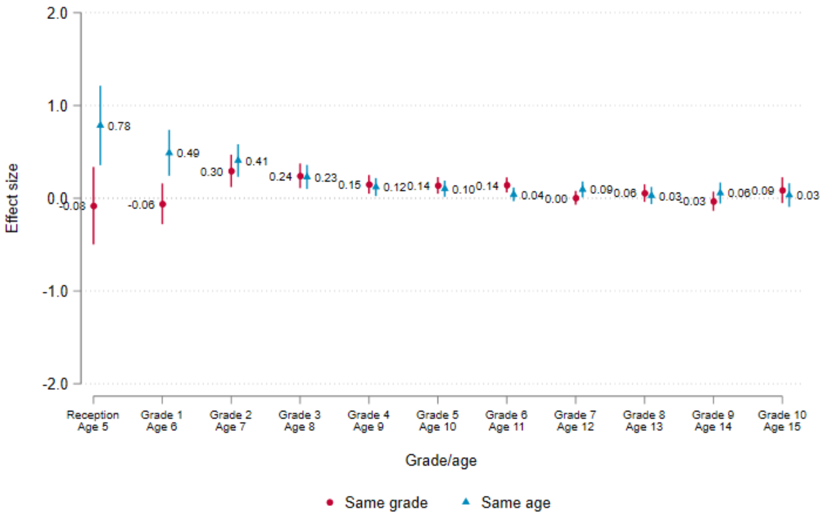

Notes: The figure plots point estimates and 95% confidence intervals of separate regressions, one for each age from 5 to 15 and grade from reception class to grade 10. The effects measure the part of the proportional increase in ADHD diagnoses explained by differences in first-time diagnoses between early and late starters (see Figure 9). In each regression, we control for sex, ethnicity, postal code SES, and maternal age at birth, and include general practice and year of birth fixed effects.

Table B.1: Effect of early start of school on ever being diagnosed ADHD

	Ever diagnosed with ADHD
	(1)
Early start	0.009***
	(0.001)
N	96,698
Mean ever being diagnosed for late starters, $Pr(EverADHDdiagnosis_{i,15} = 1 \mid Early_i = 0)$	0.017
Effect size, $r_{15} = \gamma_{15}/Pr(EverADHDdiagnosis_{i,15} = 1 \mid Early_i = 0)$	0.528

Notes: The table reports the result from regressing equation (6) on an indicator taking the value if the individual received at least one ADHD diagnosis up to age 15. The effect size is expressed as the proportional increase in the rate of having ever been diagnosed with ADHD for early starters relative to late starters, $r_{15} = \gamma_{15}/Pr(EverADHDdiagnosis_{i,15} = 1 \mid Early_i = 0)$. In each regression, we control for sex, ethnicity, postal code SES, and maternal age at birth, and include general practice and year of birth fixed effects.

Appendix C: Is the effect of starting school early different across subgroups?

We investigate whether the effect of being an early starter varies across subgroups, with particular attention to groups that prior research suggests may be less likely to be diagnosed or treated, conditional on symptoms. Specifically, we examine the impact of early school entry on ADHD pharmacological treatment for two key characteristics: gender and socioeconomic status (SES).

Figure C.1a shows that the percentage of boys receiving at least one ADHD prescription is higher than that of girls across all ages between 5 and 15. One contributing factor to this discrepancy is that ADHD symptoms in girls are more concealed than in boys. Biederman et al. (2002) compare girls and boys diagnosed with ADHD and find that girls seem to have fewer in-school and out-of-school problems and a lower likelihood of experiencing learning disabilities, and their primary ADHD symptom is inattentiveness, which is a more covert symptom than hyperactivity and impulsivity usually observed in boys. These gender differences have been interpreted in the prior literature as consistent with a higher risk of underdiagnosis and undertreatment among girls.

To examine how school-starting age affects pharmacological treatment for ADHD differently across genders, we estimate equation (6) separately for boys and girls. The results are reported in Figure C.2a in terms of the proportional increase in ADHD pharmacological treatment rate by age. The point estimates are larger for girls than for boys, but this difference is not statistically significant at any age. One interpretation is that relative age at school entry may have different implications for observed behavior and referrals by gender, potentially generating differential impacts on treatment. Given the imprecision of the estimates, we treat these subgroup differences as suggestive.

To investigate socioeconomic disparities in pharmacological treatment for ADHD, we use the Townsend deprivation score, an area-level measure of SES. As explained in Section 4, our measure of SES is provided in quintiles. We designate areas in the bottom two quintiles (i.e., the two least deprived quintiles) as high SES, while areas in the top two quintiles are classified as low SES. In Figure C.1b, we present the percentage of children with ADHD prescriptions by age, separately, for these two groups. Children living in low SES areas are more likely to be prescribed pharmacological treatment for ADHD. However, because children from low SES backgrounds are exposed to different risk factors and constraints, these raw differences do not map directly into

conclusions about under- or overtreatment.[1] Evidence on underdiagnosis of ADHD in low-SES children has been provided by Elder (2010), Emma Degroote and Houtte (2022), and Elder and Zhou (2021). Elder (2010) shows that for low-SES children (i.e., children in the bottom quartile of a composite SES index based on parental education, occupation, and income), the predicted probability of ADHD based on parents' reports is higher than the equivalent probability based on teachers' reports, which is higher than the probability based on actual diagnosis. The opposite relationship is held for children with high SES (top quartile). Emma Degroote and Houtte (2022) find that children with higher cognitive skills are more likely to be excused for their ADHD behavioral issues by providing them with an ADHD label. Because high-SES children tend to have higher cognitive skills, they are also probably more likely to be labeled as children with ADHD. Elder and Zhou (2021) suggest that children from low SES backgrounds, such as black children, are more likely to have school peers with lower skills, and because of the comparison bias, they are less likely to be diagnosed with ADHD. An overdiagnosis in children for high SES can also be explained by parents from privileged backgrounds being more aware of ADHD and more concerned about having their child treated for potential behavioral issues.

Subsequently, we separately estimate the effects of early school entry for low- and high-SES children following the same procedure as for boys and girls. The results reported in Figure C.2b illustrate that the effects of early school entry are more substantial for high-SES children. However, the confidence intervals overlap for all ages except for age 10. One interpretation is that differences in early investments and preschool environments may generate different gaps at school entry between early and late starters across SES groups (see Elder and Lubotsky, 2009), which could interact with relative-age comparisons in the classroom. Given the overlap in confidence intervals, we view these differences as suggestive rather than definitive.

Taken together, the heterogeneity patterns are consistent with the idea that relative age can interact with group-specific detection and referral processes, as emphasized in the prior literature. However, our data do not include measures of underlying ADHD symptom severity or a clinically appropriate treatment threshold. For this reason, we do not use these subgroup patterns to infer the direction of potential over- or underprescription at the individual level, and we interpret the heterogeneity results as exploratory.

We also explore whether the effect of starting school on first-time prescriptions varies by gender and SES. We define the first-time prescription rate and report the effects (see equation (9))

[1] For example, low SES children are exposed to more ADHD risk factors such as low birth weight and maternal mental depression (Saigal et al., 2003).

separately by gender and SES in Figure C.3. The results suggest that the impact of early school entry on ADHD pharmacological treatment is primarily driven by differences in initiation at younger ages, with limited evidence of new initiation effects at older ages. This pattern is consistent with persistence in treatment once prescriptions are initiated earlier in childhood.

Figure C.1: Rate of ADHD prescriptions by age: heterogeneity by gender and socioeconomic background

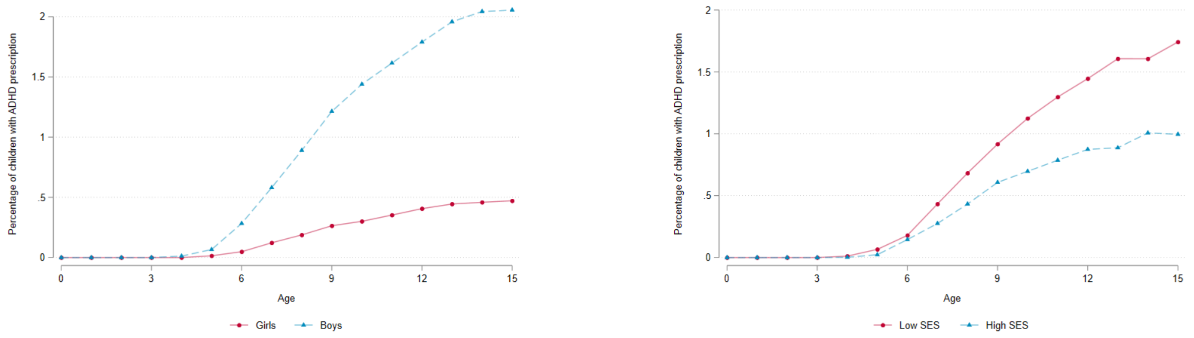

(a) By gender **(b)** By socioeconomic area background

Notes: Each marker captures the percentage of children receiving an ADHD prescription in the corresponding age period. Low (high) SES is defined as living in deprived (non-deprived) areas, as indicated by a Townsend deprivation score in the top (bottom) two quintiles.

Figure C.2: Effect of early start of school on ADHD prescriptions by age: heterogeneity by gender and socioeconomic area background

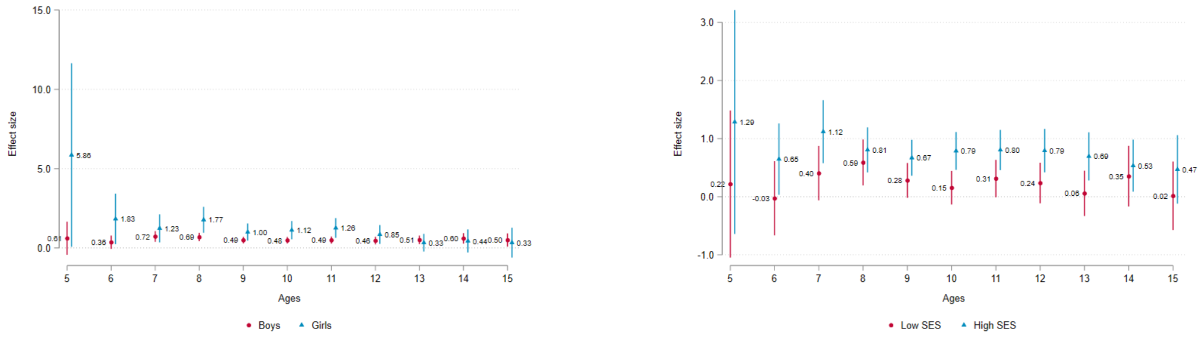

(a) By gender **(b)** By socioeconomic area background

Notes: the figure plots point estimates and 95% confidence intervals of separate regressions, one for each age from 5 to 15. Low (high) SES is defined as living in deprived (non-deprived) areas, as indicated by a Townsend deprivation score in the top (bottom) two quintiles. The effects are expressed as a proportional increase in the prescription rate for early starters relative to late starters, $r_a = \gamma_a / Pr(ADHD_{i,a} = 1 | Early_i = 0)$. In each regression, we control for sex, ethnicity, postal code SES, and maternal age at birth, and include general practice and year of birth fixed effects.

Figure C.3: Effect of early start of school on ADHD first-time prescriptions by age: heterogeneity by gender and socioeconomic area background

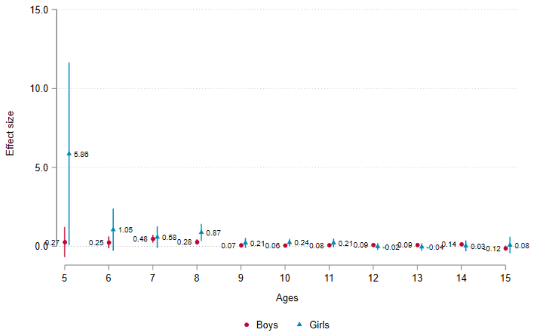

(a) By gender

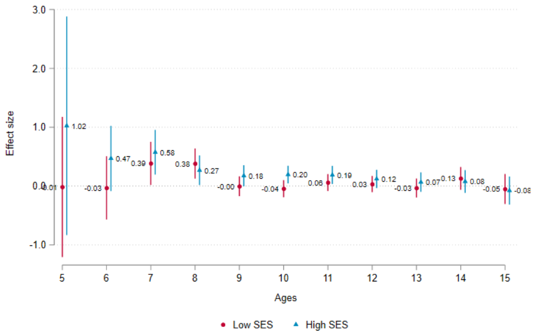

(b) By socioeconomic area background

Notes: the figure plots point estimates and 95% confidence intervals of separate regressions, one for each age from 5 to 15. Low (high) SES is defined as living in deprived (non-deprived) areas, as indicated by a Townsend deprivation score in the top (bottom) two quintiles. The effects measure the part of the proportional increase in ADHD pharmacological treatments explained by differences in first-time prescriptions between early and late starters (see equation 9). In each regression, we control for sex, ethnicity, postal code SES, and maternal age at birth, and include general practice and year of birth fixed effects.

References

Biederman, J., E. Mick, S. V. Faraone, E. Braaten, A. Doyle, T. Spencer, T. E. Wilens, E. Frazier, and M. A. Johnson (2002). Influence of gender on attention deficit hyperactivity disorder in children referred to a psychiatric clinic. *American Journal of Psychiatry 159*(1), 36–42.

Elder, T. and Y. Zhou (2021). The black-white gap in noncognitive skills among elementary school children. *American Economic Journal: Applied Economics 13*(1), 105–32.

Elder, T. E. (2010). The importance of relative standards in ADHD diagnoses: Evidence based on exact birth dates. *Journal of Health Economics 29*(5), 641–656.

Elder, T. E. and D. H. Lubotsky (2009). Kindergarten entrance age and children's achievement: Impacts of state policies, family background, and peers. *Journal of Human Resources 44*(3), 641–683.

Emma Degroote, M.-C. B. and M. V. Houtte (2022). Suspicion of ADHD by teachers in relation to their perception of students' cognitive capacities: do cognitively strong students escape verdict? *International Journal of Inclusive Education 0*(0), 1–15.

Saigal, S., J. Pinelli, L. Hoult, M. M. Kim, and M. Boyle (2003). Psychopathology and social competencies of adolescents who were extremely low birth weight. *Pediatrics 111*(5), 969–975.
